# Limb kinematics and morphology improve salamander climbing performance

**DOI:** 10.1242/jeb.251894

**Published:** 2026-06-04

**Authors:** Jonathan M. Huie, Sandy M. Kawano

**Affiliations:** ^1^ Department of Biological Sciences, The George Washington University, Washington, DC 20052, USA; ^2^ Department of Ecology and Evolutionary Biology, University of California Irvine, Irvine, CA 92697, USA; ^3^ Department of Biology, University of Virginia, Charlottesville, VA 22903, USA; ^4^ Mountain Lake Biological Station, Pembroke, VA 24136, USA

**Keywords:** Arboreal, *Aneides*, Biomechanics, Plethodontidae, Scansorial

## Abstract

Hundreds of plethodontid salamander species can climb vertical structures, despite lacking morphological adaptations typically found in other climbing tetrapods. To compensate, salamanders likely rely more on behavioral modifications to mediate the relationship between their relatively generalist morphologies and climbing performance. Here, we examined four plethodontid species (*Aneides aeneus*, *Aneides lugubris*, *Aneides hardii* and *Plethodon glutinosus*) that differ in their habitat preferences, climbing tendencies and limb morphologies. Using 3D high-speed videography, we compared how these species adjust their gait and limb kinematics while traversing a flat surface inclined at 0, 45, 80 and 90 deg. We found that all species could climb vertically (or near vertically for *A. hardii*) using similar gait and kinematic changes that increase stability. For instance, all species used a single-step gait, increased duty factor, reduced stride length and reduced stride frequency while their bodies were positioned closer to the substrate at the highest inclines compared with 0 or 45 deg inclines. However, highly scansorial species (*A. aeneus* and *A. lugubris*) climbed faster than the other species. The enhanced abilities of scansorial species may be attributable to their longer limbs that enable longer strides as well as their unique foot morphologies – coupled with changes in foot orientation – that facilitate better attachment through grasping. Overall, we propose that behavioral changes are sufficient for adequate climbing, but subtle morphological changes promote exceptional climbing performance. This likely explains the prevalence of climbing abilities across ecologically and morphologically diverse plethodontid salamanders.

## Teaser

**Summary:** Comparison of the climbing kinematics of ecomorphologically distinct salamander species shows all climb well, underscoring the role of behavior in determining performance, but habitual climbers with longer limbs and larger feet tend to climb faster.

## INTRODUCTION

Climbing is a ubiquitous behavior across animals that enables them to traverse and exploit the vertical axis of their environment. On vertical substrates, the downward force of gravity acts to dislodge animals and can be counteracted by attachment mechanisms that reduce the risk of falling (Cartmill, 1974, 1985; Preuschoft, 2002; Young, 2023). Many animals have morphological traits suited for clinging, such as claws, or have evolved novel adaptations such as adhesive toe pads (Cartmill, 1974; Labonte and Federle, 2015; Langowski et al., 2018a; Miller and Stroud, 2021; Schulz et al., 2025), and may modulate the use of these structures to increase their effectiveness (Autumn et al., 2006; Endlein et al., 2013; Sustaita et al., 2013). However, many climbing animals lack structures specifically for attachment and, in turn, may rely more on behavioral adjustments to move across inclines. Many studies have investigated how disparate animals adjust their gait and limb kinematics to increase locomotor stability while climbing, but often focus on only one or two species at a time (Jayne and Irschick, 1999; Zaaf et al., 2001a,b; Wang et al., 2014; Young et al., 2023, 2024a; Riiska et al., 2025). Identifying the relative contributions of behavior and morphology towards climbing across a range of closely related species may yield valuable insights into the evolution and minimum requirements of scansoriality.

Lungless salamanders (Plethodontidae) provide a compelling system to examine how behavior may mediate the relationship between morphology and climbing performance. Despite lacking claws or adhesive toe pads, there are hundreds of species known to climb on vegetation or rock structures (Stebbins, 1947; Gordon, 1952; Jensen et al., 2002; McEntire, 2016). Many scansorial salamanders do not have body or foot shapes distinct from those of ground-dwelling species, suggesting that specialized morphologies are not required for climbing (Blankers et al., 2012; Baken and Adams, 2019). Some species of *Bolitoglossa* have webbed feet that could improve attachment to substrates by generating suction (Alberch, 1981) but their feet lack morphological features characteristic of biological suction cups (Huie et al., 2022). Instead, modified limb kinematics coupled with mucus adhesion may be sufficient for ascending inclines as many salamanders can cling statically to vertical surfaces using the mucus layer that covers their bodies (Baken and O'Donnell, 2021; O'Donnell and Deban, 2020a,b). Nevertheless, climbing salamanders within the genus *Aneides* have morphological features hypothesized to support a scansorial lifestyle, such as long limbs that increase climbing speeds, large feet that enhance clinging performance, long digits modified for gripping and distinct carpal and tarsal arrangements purported to enhance force transmission (Wake, 1963; Larson et al., 1981; Huie et al., 2025). Because no studies to date have compared the climbing performances of multiple salamander species, it remains unclear how morphological variation may affect climbing ability.

Animals often modify their spatiotemporal gait parameters and limb posture to increase stability when ascending inclines (Zaaf et al., 2001b; Stevens, 2006; Lammers and Zurcher, 2011; Ekhator et al., 2023; Young et al., 2024a; Riiska et al., 2025). On level ground, terrestrial walking in salamanders is described as a diagonal-couplet, lateral-sequence walk, where the hindlimb and the contralateral forelimb move synchronously (Karakasiliotis et al., 2013; Pierce et al., 2020). When climbing up a broad vertical surface, the arboreal *Aneides vagrans* reduces its overall speed and uses a single-foot lateral-sequence gait, where only one forefoot or hindfoot is moved at a time (Aretz et al., 2022). Vertical climbing in *A*. *vagrans* is also characterized by increased duty factor, shorter stride length and lower stride frequency compared with level walking (Aretz et al., 2022). The terrestrial *Plethodon cinereus* makes similar adjustments to its gait when climbing on smooth surfaces but is unable to climb on a coarse surface (Hanna et al., 2022). These changes parallel those made by several species of lizards that share comparable body shapes and gaits with salamanders (Jayne and Irschick, 1999; Wang et al., 2014; Zaaf et al., 2001b). Many animals also climb with their center of mass close to the surface by crouching and increasing flexion of the limbs (Cartmill, 1985; Preuschoft, 2002; Young, 2023). By coupling a crouched posture with a wider lateral spread of their limbs, climbers can maintain stability and minimize the likelihood of falling (Chen et al., 2006; Edwards, 1977; Lammers and Zurcher, 2011; Ting et al., 1994).

Salamanders may also modify the relative functions of their limbs when climbing compared with walking. During level walking, salamanders and lizards primarily use their hindlimbs to generate propulsion and their forelimbs to brake (Autumn et al., 2006; Kawano and Blob, 2013, 2022; Wang et al., 2015). While locomotor forces have not been measured for climbing salamanders, the forelimbs of arboreal lizards take on a larger propulsatory role while climbing (Autumn et al., 2006; Chen et al., 2006; Wang et al., 2015; Munteanu et al., 2023). As propulsion in salamanders is generated through a combination of limb retraction, long-axis rotation of the proximal limb segment and lateral rotation of the girdles (Edwards, 1977), examining how limb and girdle kinematics change across different inclines may provide insights into the relative functions of the forelimbs and hindlimbs during climbing (Foster and Higham, 2012). Foot orientation and lateral spread of the digits might also change with incline angle. Arranging the feet in the fore–aft direction and narrowing digital spread to directly oppose gravity may improve attachment and propulsion (Zhuang and Higham, 2016). However, some climbing lizards and frogs increase their attachment by grasping broad surfaces, which involves rotating their feet laterally away from the midline and pulling medially with their limbs to ‘hug’ the surface (Autumn et al., 2006; Endlein et al., 2013; Zhuang and Higham, 2016). There is mixed evidence for whether salamanders have the ability to grasp (Hanna et al., 2022; O'Donnell and Deban, 2020b), but observing more lateral rotation of the feet during climbing would suggest grasping is a plausible attachment mechanism for salamanders.

**Fig. 1.**
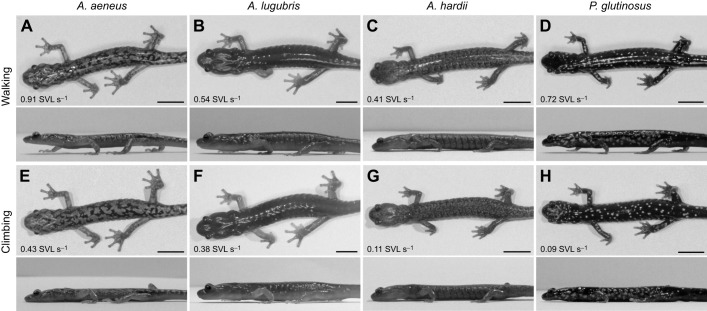
**Images of the study species during walking and climbing.** Dorsal and lateral views of (A,E) *Aneides aeneus*, 5.9 cm snout–vent length (SVL); (B,F) *Aneides lugubris*, 6.8 cm SVL; (C,G) *Aneides hardii*, 5.4 cm SVL; and (D,H) *Plethodon glutinosus*, 6.9 cm SVL. Still images from representative videos depict the animals walking on a horizontal surface (A–D) or climbing at 80 deg (G) or 90 deg (E,F,H). Scale bars: 1 cm.

In this study, we investigated how ecologically and morphologically distinct species of *Aneides* and *Plethodon* salamanders climb on different inclines (Fig. 1). Using high-speed videography, we quantified the three-dimensional (3D) kinematics of the limbs and spatiotemporal gait parameters during walking and climbing. We aimed to investigate whether the climbing kinematics and abilities of closely related species vary based on scansorial ecology and concomitant morphological differences. We predicted that all four species in this study make similar changes to their gait and limb kinematics that result in slower organismal speeds, keeping their bodies close to the surface, widening their grip and rotating their feet more laterally. However, we anticipated that species with longer limbs and larger feet would climb faster. We also predicted that forelimb and hindlimb kinematics would be modified in different ways during climbing versus walking, reflecting their different locomotor functions. We found evidence that behavior can mediate the relationships between generalized morphologies and climbing performance, but the possession of certain morphological features can promote exceptional climbing abilities.

## MATERIALS AND METHODS

### Specimen sampling

We studied four species [*Aneides aeneus* (Cope & Packard 1881), *Aneides lugubris* (Hallowell 1849), *Plethodon glutinosus* (Green 1818) and *Aneides hardii* (Taylor 1941)] that, in decreasing order, represent a morphological gradient ranging from relatively long limbs with large feet to relatively short limbs with small feet (Huie et al., 2025) (Fig. 1). These species also have microhabitat preferences associated with different climbing tendencies. *Aneides aeneus* is highly scansorial and spends most of its life above ground on rock outcrops (Gordon, 1952). *Aneides lugubris* is moderately scansorial and semi-arboreal, as individuals can be found in tree holes and stumps or on the ground (Ritter and Miller, 1899; Miller, 1944). Meanwhile, *P. glutinosus* is a terrestrial generalist often found on the ground but will occasionally climb on rock structures, making it mildly scansorial (Jensen et al., 2002; Marshall et al., 2004). In some areas, *A. aeneus* and *P. glutinosus* are syntopic and will stratify the same rock outcrops but *A. aeneus* consistently climbs higher than *P. glutinosus* (Cliburn and Porter, 1987; Waldron, 2000), suggesting that the former has better climbing ability. Lastly, the terrestrial *A. hardii* does not normally climb and is often found under or within logs (Scott and Ramotnik, 1992).

Between 2022 and 2024, we collected specimens of *A. aeneus* (*n*=10) and *P. glutinosus* (*n*=10) from West Virginia and Kentucky, *A. lugubris* (*n*=10) from California, and *A. hardii* (*n*=10) from New Mexico (see Table 1). Specimens of *P. glutinosus* were collected from the same localities and collecting trips as *A. aeneus*. Animals were individually housed in enclosures with humidity levels of 80–100% and temperatures of 15–20°C. Animals were fed crickets, bean weevils or earthworms up to twice per week. Collecting permits were granted by the relevant state wildlife agencies (CDFW #40628875-8, KDFWR #2211267 and #2311307, NMDGF #3861, USFWS #MAPER0045444, WVDNR #2022.314 and #2023.255) and experimental and animal care procedures were approved by the Institutional Animal Care and Use Committee at The George Washington University (protocols #A2020-022 and #A2023-047).

**Table 1.** Specimen information and sample sizes

Species	*Aneides aeneus*	*Aneides lugubris*	*Aneides hardii*	*Plethodon glutinosus*
No. of individuals	10	10	10	10
Snout–vent length (cm)	4.61±0.24 (3.7–6.0)	5.35±0.52 (2.9–7.6)	4.82±0.20 (3.9–5.8)	5.12±0.36 (3.4–6.9)
Mass (g)	1.50±0.21 (0.8–3.1)	3.55±0.95 (0.4–8.9)	1.68±0.24 (0.8–3.2)	2.66±0.61 (0.5–6.3)
Forelimb length (cm)	0.94±0.04 (0.75–1.15)	0.97±0.07 (0.61–1.29)	0.75±0.02 (0.66–0.87)	0.91±0.04 (0.68–1.13)
Hindlimb length (cm)	0.98±0.04 (0.80–1.18)	1.03±0.08 (0.64–1.43)	0.78±0.02 (0.69–0.92)	0.96±0.05 (0.71–1.18)
Forefoot area (cm^2^)	0.11±0.01 (0.07–0.17)	0.15±0.02 (0.05–0.29)	0.07±0.01 (0.05–0.11)	0.09±0.01 (0.04–0.14)
Hindfoot area (cm^2^)	0.19±0.02 (0.12–0.32)	0.26±0.08 (0.08–0.50)	0.13±0.01 (0.09–0.18)	0.17±0.02 (0.07–0.26)
No. of trials (0, 45, 80, 90 deg)	50, 50, 50, 50	50, 50, 46, 36	50, 50, 50, 3	50, 50, 50, 50

The raw trait values are presented as means±s.e. with the range of values in parentheses.

### Collection of locomotor trials

Locomotor trials were recorded simultaneously in the dorsal and lateral views using two synchronized high-speed cameras (VEO 340S, Vision Research Inc.) at 400 frames s^−1^ and with an exposure time of 500 μs. Two sets of camera lenses were used depending on the size of the animal; either Canon Ultrasonic 50 mm lenses or Canon EF 100 mm lenses. Individuals were recorded moving on a flat piece of acrylic oriented at four inclines (0, 45, 80 and 90 deg). The acrylic was covered with a thin layer of vinyl with surface asperities that ranged between 200 and 580 μm in diameter, which were measured from a macro photo using FIJI v2.3 (Schindelin et al., 2012). The filming arena was composed of a 20-gallon (∼76 l) glass aquarium illuminated by a combination of LED light panels, LED spotlights and infrared spotlights. Each animal was given at least a 10 min rest period between trials and filmed on only one incline per day. Animals were also fasted for at least 2 days prior to experimentation to avoid the effects of satiety (Sass and Motta, 2002).

3D kinematics were analyzed from 735 trials (Table 1). Within each trial, we analyzed a single hindlimb stride (typically a middle stride) and the following ipsilateral forelimb stride. We collected trials that represented volitional locomotor speeds because that is more likely to reflect the habitual movements used by animals in their environments, whereas maximum sprint speeds are observed during escape responses that are used less frequently (Irschick, 2000). Thus, we sparingly encouraged individuals to move but did so by gently tapping their tails and placing a small shelter at the far end of the trackway. For quality control, the trials used for subsequent analyses required that the individual complete at least three continuous stride cycles in a single direction at a relatively steady speed. We attempted to collect five trials per individual on each incline, but some individuals would not climb continuously or at all (especially at 80 and 90 deg inclines). If the climbing bout was discontinuous, we retained trials that captured at least one hindlimb stride and the ipsilateral forelimb stride. We also recorded videos of calibration checkerboards to convert our data from 2D coordinates to 3D coordinates. Calibration checkerboards were generated with the *stereomorph* R package v1.6.1 (Olsen and Westneat, 2015) and filmed periodically throughout each filming session. These videos were used to reconstruct the camera positions, correct for image distortion and generate camera calibration matrices in XMALab v2.1 (Knörlein et al., 2016).

### Semi-automated tracking of anatomical landmarks

**Fig. 2.**
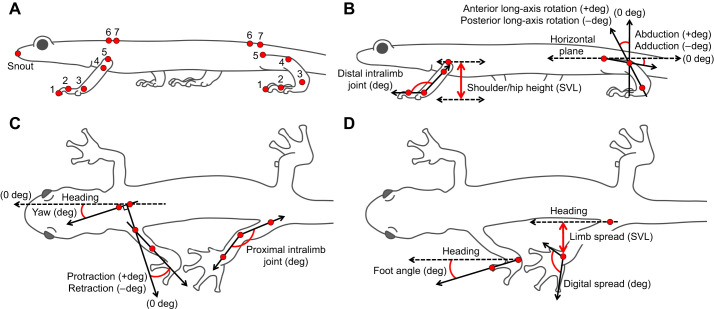
**Anatomical landmarks and schematics of the kinematic variables.** (A) The snout and seven homologous landmarks on the forelimbs and hindlimbs were tracked throughout each trial: (1) the tip of the longest digit of the forefoot/hindfoot, (2) the metacarpophalangeal/metatarsophalangeal joint, (3) the wrist/ankle, (4) the elbow/knee, (5) the shoulder/hip, and (6,7) two points along the midline of the body that were dorsal to the pectoral/pelvic girdle. (B) Visual representations of the distal intralimb joint angle, shoulder/hip height, limb abduction/adduction relative to a horizontal plane at the height of landmark 5, and long-axis rotation of the proximal limb segment. (C) Visual representations of girdle rotation (yaw) relative to animal's heading, limb protraction/retraction relative to an axis orthogonal to the midline, and the proximal intralimb joint angle. (D) Visual representations of foot angle and lateral limb spread relative to the animal's heading as well as digital spread.

The 3D position of seven anatomical landmarks was tracked throughout each trial on the forelimb and hindlimb, following the landmarking scheme of Kawano et al. (2016). These included: (1) the tip of the longest digit of the forefoot/hindfoot, (2) the metacarpophalangeal/metatarsophalangeal joint, (3) the wrist/ankle, (4) the elbow/knee, (5) the shoulder/hip, and (6,7) two points along the midline of the body that were directly dorsal to the anterior and posterior margins of the pectoral/pelvic girdle (Fig. 2A). We used XMALab to manually track points for 1948 dorsal and lateral frames sampled from 65 trials to generate a training dataset for DeepLabCut, a toolbox for markerless pose-estimation (Mathis et al., 2018). DeepLabCut v2.3.8 was used to train a single ResNet-50 neural network with the default parameters for 500,000 iterations. The resulting network had an average testing error of 2.99 pixels (∼0.03 mm). The network was trained with 10 additional landmarks at the tip of the snout and along the midline of the trunk and tail to improve tracking performance, but the tip of the snout was the only landmark out of these additional 10 that was included in the analyses of this study. We used the network to analyze the videos for all 735 trials, including the trials used to make the training data as we only manually tracked a portion of those trials.

Our workflow was facilitated by several custom Python scripts. To extract training frames and analyze new videos, we modified the code from Laurence-Chasen et al. (2020), which integrates XMALab with DeepLabCut, and adapted it for larger datasets. We also wrote scripts to extract the camera calibration matrices from XMALab files and convert the 2D coordinates predicted by DeepLabCut into 3D coordinates. All scripts used for this study are available from GitHub (https://github.com/jmhuie/Salamander_Climbing_Kine).

### Spatiotemporal gait parameters

We first quantified four spatiotemporal gait parameters that other salamander species modulate to increase climbing stability (Aretz et al., 2022; Hanna et al., 2022). The gait parameters included: (1) whole-organism speed, (2) duty factor, (3) stride length and (4) stride frequency. Speed was calculated as the distance traveled by the tip of the snout from the beginning of the hindlimb stride until the end of the ipsilateral forelimb stride, represented as the number of snout–vent lengths (SVLs) traveled forward per second. Duty factor was calculated as the proportion of a stride cycle that was spent during the stance phase (i.e. when the forefoot or hindfoot of the focal limb was in contact with the surface). Stride length was calculated as the distance traveled by the wrist or ankle landmark throughout a stride cycle and was represented as a proportion of SVL. Stride frequency was calculated as the reciprocal of stride duration. Identification of the stance and swing phases and calculation of stride duration were done by manually inspecting the videos in FIJI.

### Angular and linear kinematics

To characterize the movements of the limbs and girdles, we calculated 10 angular and linear kinematic variables using the anatomical landmarks (Fig. 2). The angular kinematics included: (1) abduction versus adduction and (2) protraction versus retraction of the proximal limb segments about the shoulder/hip joint; (3) long-axis rotation of the proximal limb segments; flexion versus extension of (4) the proximal intralimb joint (elbow/knee) and (5) the distal intralimb joint (wrist/ankle); (6) yaw of the pectoral/pelvic girdle; (7) orientation of the forefeet/hindfeet relative to the animal's heading; and (8) the lateral spread of the digits of the forefeet/hindfeet. Linear kinematics were represented by (9) the lateral spread of the forelimbs and hindlimbs, and (10) the vertical height of the shoulder/hip joint. Most of these variables were calculated in 3D, except for digital spread, which was calculated in 2D using the dorsal videos. Calculations of protraction versus retraction, yaw, orientation of the feet, and limb spread were all done with the 3D landmarks, but were restricted to movements that occurred within the horizontal plane by projecting the relevant landmarks onto the horizontal plane and eliminating dorsoventral variation prior to angle/distance calculations. All 10 of the kinematic variables are time dependent and vary over the course of a stride cycle. Therefore, to facilitate comparisons, we summarized most of the variables (1–6 and 10) by calculating the maximum, minimum and total excursion (maximum minus minimum) value for each stride. Mean values were calculated for the foot orientation, limb spread and digital spread (7–9) within the stance phase of the stride.

Abduction and adduction refer to the movement of the proximal limb segments (i.e. humerus or femur) away from (elevation) and towards (depression) the ventral midline, respectively (Fig. 2B). We calculated the angle between the proximal limb segment and a horizontal plane at shoulder/hip height, where values of 0 deg meant the proximal limb was parallel to the ground. Positive and negative values correspond to abduction and adduction, respectively. Protraction and retraction refer to the anterior and posterior movements of the proximal limb segments in the horizontal plane, respectively. We calculated the angle between the proximal limb segment and a mediolateral axis that was orthogonal to the dorsal midline points above each girdle. Values of 0 deg mean the proximal limb was perpendicular to the midline, while positive and negative values correspond to protraction and retraction, respectively (Fig. 2C). Long-axis rotation of the limb refers to anterior and posterior rotation of the proximal limb segment. We calculated the angle between a plane containing the proximal and distal limb segments and a vector perpendicular to the ground, while accounting for variation in limb abduction and adduction. Values of 0 deg mean the proximal limb was aligned with the vertical axis, while positive and negative values correspond to greater anterior and posterior rotation, respectively (Fig. 2B).

The elbow/knee joint and wrist/ankle joint were considered flexed with angular values less than or equal to 90 deg, and extended with values greater than 90 deg (Fig. 2B,C). Yaw was calculated as the angle between the midline points and a vector representing the animal's heading, describing the amount of lateral rotation exhibited by the girdle. Positive values indicate rotation of the girdle towards the contralateral side, and negative to the ipsilateral side (Fig. 2C). Foot orientation during stance was calculated as the angle between a vector defined by the tip of the longest (third) finger/toe and the wrist/ankle joint and a vector representing the animal's heading. Values of 0 deg mean the foot was pointed forward in the direction of the heading, while positive and negative values indicate that the feet were pointed away from and towards the midline, respectively (Fig. 2D). Digital spread was a 2D angle calculated in FIJI using videos from the dorsal perspective. It was calculated during stance as the angle between the tip of the first and last digits on the forefeet/hindfeet, with the wrist/ankle as the vertex (Fig. 2D).

Limb spread was calculated as the perpendicular distance between the wrist/ankle joint and a vector passing through the shoulder/hip joint representing the animal's heading during stance. Measures of limb spread were represented as a proportion of SVL (Fig. 2D). Hip and shoulder height were calculated throughout the entire stride cycle as the shortest vertical distance between the surface and the shoulder/hip joint. Hip and shoulder height were also represented as proportions of SVL (Fig. 2D).

**Fig. 3.**
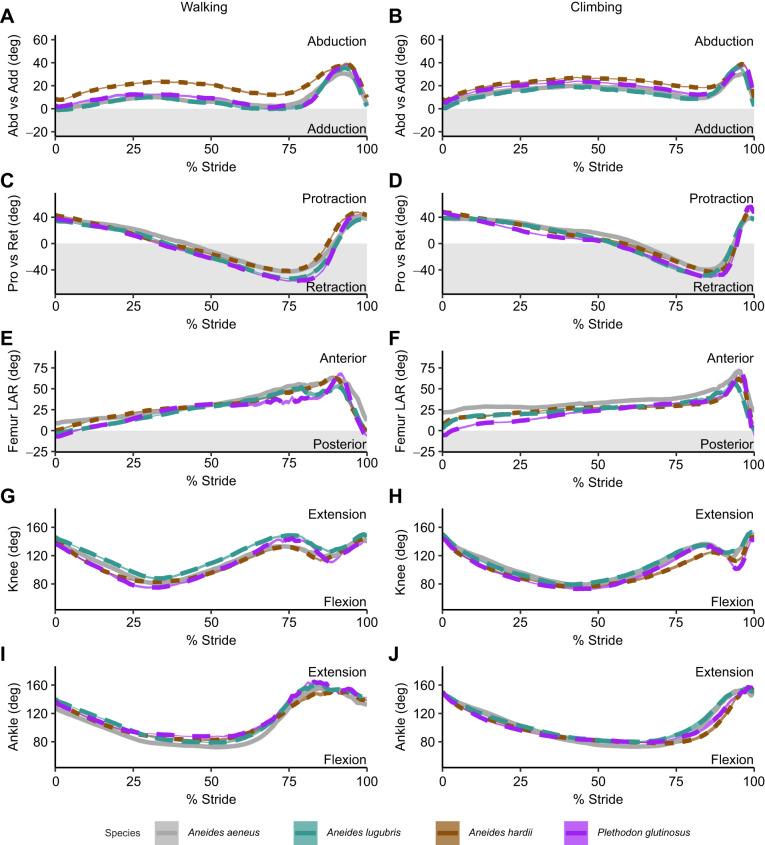
**Profiles of hindlimb kinematics during walking and climbing.** (A,B) Abduction (Abd) versus adduction (Add) of the limbs, (C,D) protraction (Pro) versus retraction (Ret) of the limbs, (E,F) long-axis rotation (LAR) of the femur, (G,H) extension and flexion of the knee, and (I,J) extension and flexion of the ankle. Individual trials were pooled for each species and profiles are plotted as means±s.e. Because of the small sample size for 90 deg (*n*=3), *A. hardii* climbing trials depict locomotion on 80 deg incline (*n*=50).

We used a combination of new R scripts and modified code from the *kraken* GitHub repository (https://github.com/MorphoFun/kraken) to calculate most kinematic variables. The code used to calculate long-axis rotation in 3D was modified from Iijima et al. (2023). Many of the joint calculations required the definition of a horizontal plane that was oriented parallel to the surface. However, our cameras were not perfectly orthogonal during filming, so the plane was not automatically reflected in the *XYZ* coordinates of the landmarks. Thus, we manually defined a horizontal plane for each set of calibration matrices using three points where the animals contacted the surface. Prior to angular calculations, the 3D coordinates for all landmarks were interpolated to 101 points (0%–100%, with each point representing 1% of the stride), using the ‘interpolateR’ function in *kraken*. Doing so allowed direct comparisons of the time-dependent angles across stride cycles with different durations (Fig. 3). All analyses were performed in R v4.4.1 (https://www.r-project.org/).

### Morphological variation

Additionally, we measured the limb lengths and surface areas of the feet for each specimen following Huie et al. (2025). Briefly, forelimb and hindlimb lengths were calculated as the sum of the proximal and distal limb segments based on the 3D anatomical landmarks. Forefoot and hindfoot area were measured by tracing the perimeter of the feet in FIJI using dorsal images from the videos (one per individual). The enclosed area of the perimeter was used as the foot surface area. Limb length and foot area were normalized to body size and represented as a proportion of SVL or the square of SVL, respectively.

### Statistical analyses

We used linear mixed-effect models (LMMs) to compare the effects of inclines and species on the spatiotemporal gait parameters and limb kinematics. We used the summarized kinematic variables (maximum, minimum and excursion for most variables but mean values for foot angle, digital spread and limb spread) as individual response variables. Our basic model structure included incline, species, limb type and their interactions as the fixed effects, as well as the identity of the individuals as the random effect to account for repeated measures. Because variation in body size often affects locomotor kinematics (Birn-Jeffery and Higham, 2014; Clemente and Dick, 2023), and was confirmed to be the case for many of our kinematic variables, we also included log(SVL) as a fixed effect. The LMMs [variable∼Incline * Species * Limb+log(SVL)+(1|ID)] were performed with random intercepts and fitted using Restricted Maximum Likelihood through the ‘lme’ function in the *nlme* R package v3.1 (https://CRAN.R-project.org/package=nlme). Only the LMM comparing locomotor speed omitted limb as a fixed effect [variable∼Incline * Species+log(SVL)+(1|ID)] as we were concerned with the overall speed of the whole organism rather than individual limbs.

To assess which fixed effects and interactions explained significant amounts of variation (Table S1), we performed *F*-tests on each of the factors by passing the LMMs through the ‘anova’ function in the *stats* package v4.4.1 (https://www.r-project.org/) (Table S1). We also assessed how much variation in the data was explained by each LMM by calculating the coefficient of determination (*R*^2^). The marginal *R*^2^ (*R*^2^_marg_) describes the amount of variation explained by only the fixed effects, while the conditional *R*^2^ (*R*^2^_cond_) describes the amount of variation explained by both the fixed and random effects. Both coefficients were calculated with the ‘r2_nakagawa’ function in the *performance* R package v0.13 (Lüdecke et al., 2021). To directly compare point estimates of the gait parameters and kinematic variables, we calculated the estimated marginal means (EMMs) and variation around the mean for each combination of the categorical fixed effects (incline, species, limb). All EMMs were calculated assuming a constant SVL of 4.94 cm, the average body size of our specimens, using the *emmeans* R package v1.10 (https://CRAN.R-project.org/package=emmeans). We reported values as EMM±standard error (s.e.) (Table S2).

We used a linear discriminant function analysis (DFA) to explore which of the kinematic variables best differentiate stride cycles by incline, species and limb. We used the summarized kinematic variables from all 735 trials as the discriminators and each combination of the categorical fixed effects as the groups. To prepare the variables for the DFA, we took the absolute value of all angles, log-transformed them, and then scaled and centered them with the ‘scale’ R function (https://www.r-project.org/). The DFA was performed using the *MASS* R package v7.3 (https://CRAN.R-project.org/package=MASS). We used the coefficients of linear discriminants (Table S3) and a biplot of the first two discriminant function axes to identify the variables associated with differentiating stride cycles between the fixed effects. Because there was substantial overlap between locomotor kinematics on 0 and 45 deg inclines as well as 80 and 90 deg, we focused our results on the differences between horizontal walking and vertical climbing. One exception is *A. hardii*, which only produced three trials on the 90 deg incline, so we focused on how it climbed on an 80 deg incline.

## RESULTS

The LMMs and associated *F*-tests indicated that most spatiotemporal gait parameters and all kinematic variables were significantly influenced by differences between incline angle and species as well as their interaction (*P*≤0.05) (Table S1). Duty factor was the only parameter that did not vary significantly with species alone (*F*=1.66, *P*=0.194) but was influenced by all other fixed effects, including interactions with species (*P*≤0.02). In general, hindlimbs and forelimbs exhibited significantly different kinematics that varied with incline and between species (*P*≤0.05). However, stride length and stride frequency did not vary between limbs or with any interactions that included limb type (*P*≥0.128). Finally, size represented by log(SVL) had a significant effect on all gait parameters and most kinematic variables but notably not the kinematics of the wrist/ankle joint (*P*≥0.685).

### Spatiotemporal gait parameters

**Fig. 4.**
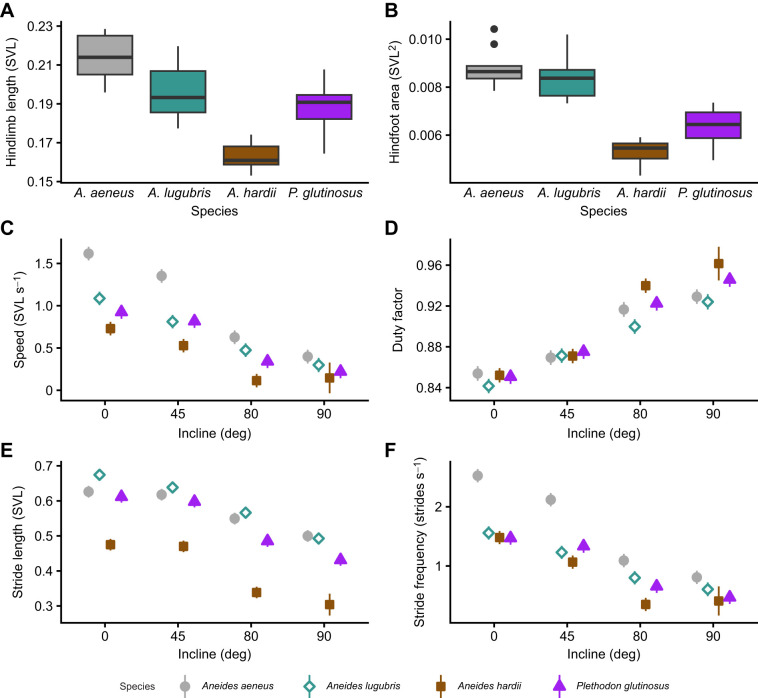
**Hindlimb morphology and gait parameters across species and inclines.** Boxplots show variation in relative (A) hindlimb length and (B) hindfoot area. Boxplots depict the median, upper and lower quartiles, interquartile range and outliers as determined by the 1.5 interquartile rule (circles). Plots of the estimated marginal means and standard error bars show variation in (C) locomotor speed, (D) duty factor, (E) stride length and (F) stride frequency.

Climbing was associated with slower locomotor speeds, increased duty factors, shorter stride lengths and lower stride frequencies compared with horizontal walking (Fig. 4; Table S2). All species walked with a diagonal-couplet, lateral-sequence gait, where the forelimbs and hindlimbs on opposite sides moved in tandem. When climbing, species switched to a single-footed lateral-sequence walk, where hindlimb footfalls were followed by the ipsilateral forelimb. Compared with species with shorter limbs, those with longer limbs and larger feet climbed faster and experienced lower reductions in speed when climbing compared with walking (Fig. 4). For instance, *A. aeneus*, *A. lugubris*, *P. glutinosus* and *A. hardii* experienced 67%, 70%, 76% and 85% reductions in speed during climbing compared with walking, respectively. *Aneides aeneus* climbed the fastest (0.44±0.08 SVL s^−1^) and moved 1.5 times faster than *A. lugubris*, 2.2 times faster than *P. glutinosus* and 4.5 times faster than *A. hardii*. Similarly, species with longer limbs took larger strides and more strides per second. Longer-limbed species also displayed smaller increases in duty factor when climbing than those with shorter limbs (Fig. 4). All spatiotemporal gait parameters were similar between the forelimbs and hindlimbs within a species (Table S2).

### Major axes of limb kinematics

**Fig. 5.**
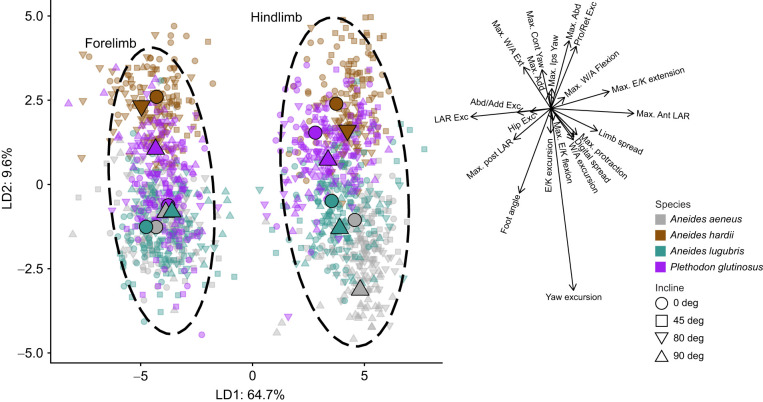
**Results of the discriminant function analysis showing separation of limb kinematics by limb, species and incline.** Non-overlapping 95% confidence ellipses highlight the separation of forelimb and hindlimb kinematics. Large circles and triangles depict the average walking and climbing, respectively, of the forelimbs and hindlimbs for each species. The biplot on the right shows the variables and their correlations with the discriminant function axes. Three variables (maximum retraction, maximum hip height and minimum hip height) were removed from the biplot to aid readability and because they held low explanatory power. Abd, abduction; Add, adduction; Ant, anterior; Cont, contralateral; Exc, excursion; Ext, extension; Flex, flexion; E/K, elbow/knee; Ips, ipsilateral; LAR, long-axis rotation; Post, posterior; Pro, protraction; Ret, retraction; W/A, wrist/ankle.

Limb kinematics were distinguishable with a DFA by limb type and, to a lesser extent, by species and incline (Fig. 5; Table S3). The first axis of the DFA (LD 1) explained 64.7% of the between-group variance and separated the forelimb and hindlimb kinematics of all species. The forelimbs were associated with larger excursions of long-axis rotation in the humerus compared with the femur, more externally rotated forefeet than hindfeet, greater extension of the wrist compared with the ankle, and larger excursions of shoulder height compared with hip height. In contrast, the hindlimbs displayed greater anterior long-axis rotation of the proximal segment, larger protraction–retraction excursions, greater extension of the knee, and were spread further than the forelimbs. The second axis (LD 2) explained 9.6% of the between-group variance and reflected differences between climbing and non-climbing species. Along LD 2, the forelimb and hindlimb kinematics of *A. aeneus* and *A. lugubris* were generally distinct from those of *A. hardii*. The latter displayed narrower girdle excursions, less lateral rotation of the feet, greater extension of the distal intralimb joint (wrist/ankle) and greater abduction of the limbs. The forelimb kinematics of *A. aeneus* and *A. lugubris* were largely indistinguishable, but *A. aeneus* displayed less abduction of their hindlimbs and less ankle extension than *A. lugubris.* Meanwhile, *P. glutinosus* displayed forelimb kinematics that were intermediate between those of the climbing and non-climbing species of *Aneides* but hindlimb kinematics that overlapped with those of *A. hardii*. Variation along LD 2 and LD 3 (7.4% of the between-group variance) also reflected differences between walking and climbing kinematics. In general, all species rotated their feet further away from their midline and increased limb abduction when climbing.

### Changes in body posture

**Fig. 6.**
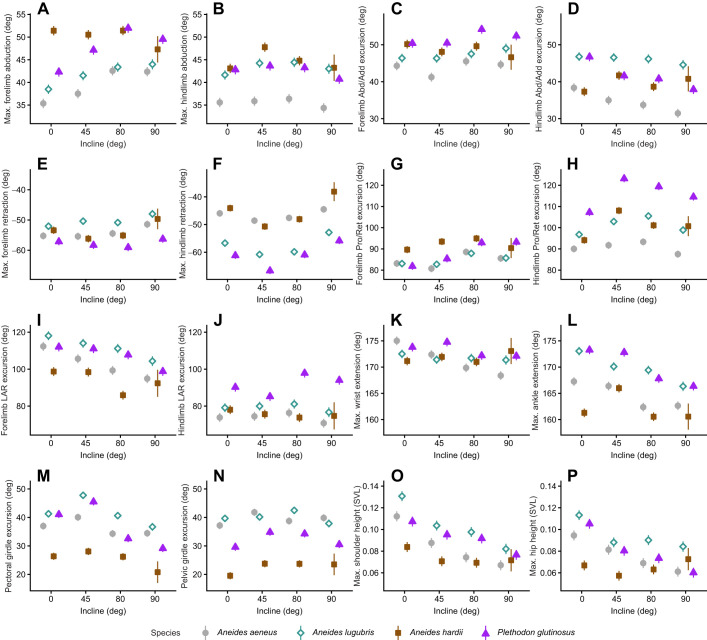
**Comparison of kinematic variables related to posture and propulsion.** Forelimb and hindlimb plots of the estimated marginal means and standard error bars show variation in (A,B) maximum abduction angle, (C,D) abduction/adduction excursion, (E,F) maximum limb retraction, (G,H) protraction/retraction excursion, (I,J) long-axis rotation of proximal limb segment excursion, (K,L) maximum extension of the distal intralimb joints, (M,N) girdle excursion and (O,P) maximum shoulder/hip height. Abd, abduction; Add, adduction; LAR, long-axis rotation; Pro, protraction; Ret, retraction.

Most species walked using a sprawled posture, with their proximal limb segments close to parallel with the surface, but *A. hardii* walked with a more crouched posture and with their venter close to the surface. When climbing, all species adopted a crouched posture and often rested their venters on the surface (Figs 1 and 6). These differences are reflected in smaller maximum shoulder and hip heights as well as narrower excursions when climbing compared with walking. The scansorial species of *Aneides* and *P. glutinous* dropped their maximum shoulder/hip height by 27–45% when climbing, while *A. hardii* made smaller changes (12–14%) (Table S2). As a result of crouching, the limbs remained more abducted for a larger proportion of the stride cycle when climbing compared with walking (Fig. 3). During climbing, maximum abduction values in the forelimbs were lower for *A. aeneus* (43.5±0.9 deg) and *A. lugubris* (43.9±1.2 deg) compared with *A. hardii* (51.7±1.0 deg) and *P. glutinosus* (50.0±1.0 deg), but were generally 5–8 deg higher for a given species (except in *A. hardii*) than during walking (Table S2). Also, the maximum abduction of the hindlimbs during climbing was lower in *A. aeneus* (33.1±0.9 deg) than in all other species (41–45 deg) (Fig. 6; Table S2). Maximum abduction values in the hindlimbs did not vary greatly with incline, but abduction/adduction excursions generally decreased on steeper inclines for all species (Fig. 6; Table S2). Climbing was also associated with changes in the flexion of the elbow, knee, wrist and ankle (Table S2), which remained flexed for a larger proportion of the stride cycle during climbing (Fig. 3).

### Kinematics related to forward displacement

Climbing was associated with increases in maximum protraction angles and decreases in maximum retraction angles for both limbs (Fig. 6; Table S2). On average, maximal protraction increased by 3–12 deg in the forelimbs and the hindlimbs during climbing across species, except for the hindlimb protraction of *A. aeneus*, which decreased by 1 deg (Table S2). Meanwhile, maximal retraction decreased by 1–5 deg in the forelimbs and hindlimbs of species besides *A. hardii*, where maximal retraction increased by 2–4 deg. All limbs remained in a protracted state for a longer portion of the stride cycle during climbing compared with walking (Fig. 3). Additionally, climbing was associated with reductions in maximum long-axis rotation of the humerus in the anterior direction and its excursion, but relatively small increases in the anterior long-axis rotation of the femur. On average, maximal anterior long-axis rotation of the humerus and excursions decreased by 12–18 deg, while maximal anterior long-axis rotation of the femur increased by 1–6 deg but excursions decreased by 2–4 deg in *Aneides* species and increased by 3 deg in *P. glutinosus* (Table S2). Climbing also involved reductions in the excursion of the pectoral girdle (0–9 deg), while excursions of the pelvic girdle remained generally unaffected (Fig. 6; Table S1). During climbing, scansorial *Aneides* species had broader excursions of both the pectoral (35–37 deg) and pelvic girdles (38–40 deg) compared with *A. hardii* and *P. glutinosus* (pectoral: 26–29 deg; pelvic: 24–30 deg) (Table S2).

### Limb adjustments related to grasping

**Fig. 7.**
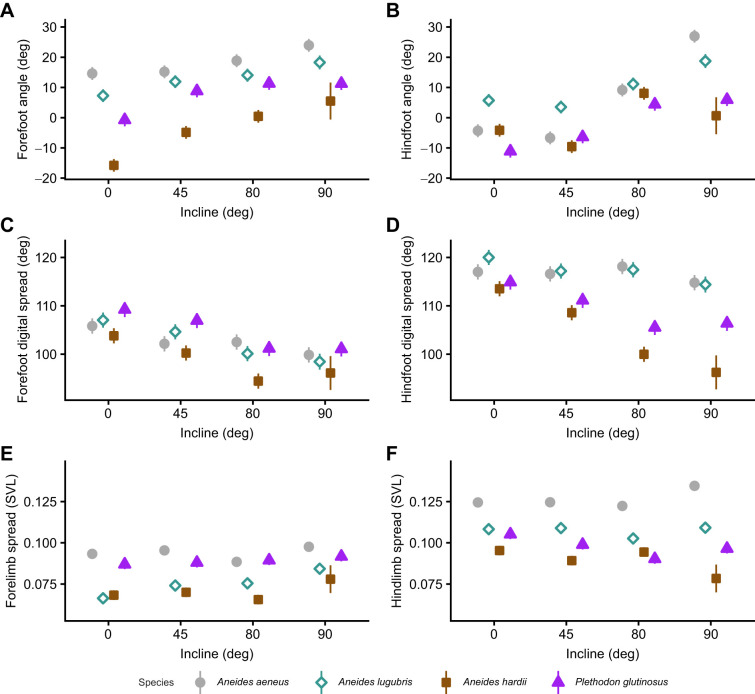
**Comparison of foot angle, digital spread and limb spread.** Plots of the estimated marginal means and standard error bars show (A,B) forefoot and hindfoot angle relative to the heading, (C,D) forefoot and hindfoot digital spread, and (E,F) forelimb and hindlimb spread.

All species rotated their feet laterally at the wrist/ankle joint and pointed them further away from the midline when climbing compared with walking (Fig. 7; Table S2). During walking, most feet were rotated 1–16 deg towards the midline, except for the forefeet of *A. aeneus* and both feet of *A. lugubris*, which were pointed away from it (6–15 deg). When climbing, the scansorial species of *Aneides* rotated their feet more laterally (18–27 deg) compared with the other species, which pointed their feet forward (0 deg) or rotated them up to 11 deg away from the midline.

The salamanders also decreased the angle between their first and last digit, or the spread of their digits, on each foot when climbing compared with walking (Fig. 7; Table S2). On average, all species walked with their fingers spread 103–109 deg and climbed with their fingers 6–11 deg closer together. However, the scansorial *Aneides* species walked and climbed with similar digital spreads in their hindfeet (117–120 deg versus 114 deg), while the other species walked with average digital spreads of 113–114 deg but climbed with narrower digital spreads of 99–106 deg.

Meanwhile, most species did not change how far they spread their limbs when locomoting on inclined surfaces. Only *A. lugubris* increased the lateral spread of their forelimbs when climbing compared with walking, by an average of 34% (Fig. 7; Table S2). *Aneides aeneus* widened their hindlimb spread by a similar amount, whereas *A. lugubris* widened their hindlimb spread by only 10%. Neither *A. hardii* nor *P. glutinosus* widened their limb spread on inclines (Fig. 7). Moreover, interspecific differences in limb spread did not match relative differences in limb length. For instance, *P. glutinosus* has proportionally longer hindlimbs than *A. hardii* but the two species spread their hindlimbs a similar proportion. That suggests *A. hardii* spread their hindlimbs unusually far, *P. glutinosus* keep their hindlimbs close to their body, or some combination of these scenarios. Similarly, *A. lugubris* has proportionally longer limbs than *P. glutinosus* but the latter spread their forelimbs relatively further on most inclines.

## DISCUSSION

We found that scansorial salamanders, which have longer limbs and larger feet, are better vertical climbers than habitual ground-dwelling species. All species of *Aneides* and *Plethodon* that we examined can climb vertical or near-vertical surfaces, which is likely aided by modulation of their gait and limb kinematics. For instance, they all change to a single-step gait, increase duty factor, reduce stride length, reduce stride frequency and position their bodies closer to the surface. These adjustments increase locomotor stability and parallel those made by other climbing salamanders (Aretz et al., 2022; Hanna et al., 2022), lizards (Jayne and Irschick, 1999; Zaaf et al., 2001a,b; Wang et al., 2014) and frogs (Young et al., 2023). However, species with relatively longer limbs and larger feet climb faster and experience a smaller reduction in locomotor speed while climbing compared with species with shorter limbs. These patterns support the hypothesis that the distinct morphological characteristics of scansorial species of *Aneides* are adaptations for climbing (Huie et al., 2025; Larson et al., 1981). Thus, we propose that behavioral flexibility is sufficient to climb nearly vertical structures, but morphological traits that improve stability and attachment promote exceptional climbing abilities.

The climbing ability of *A. hardii* is impressive, considering its primarily terrestrial lifestyle and unremarkable limb morphology (i.e. short limbs and small feet), which lacks clear advantages for climbing. This species exemplifies how modulating kinematics can expand locomotor capabilities without requiring morphological changes. However, there appears to be a functional limit to behavioral mediation. *Aneides hardii* is largely unable or unwilling to climb vertically, evidenced by our small sample size, suggesting that it cannot generate sufficient attachment forces to resist gravity and shear. *Plethodon cinereus*, another short-legged and small-footed salamander, is also unable to climb vertically on a broad coarse surface but can climb a smooth surface (Hanna et al., 2022). The inability of *A. hardii* and *P. cinereus* to climb coarse vertical surfaces is most likely attributable to small surface irregularities that disrupt their mucosal adhesion (O'Donnell and Deban, 2020b; Hanna et al., 2022). Salamanders use the mucus layer that covers their venter to increase clinging performance (O'Donnell and Deban, 2020a,b), and our observations suggest it may also be used to increase climbing performance (Figs 1 and 6). The resulting surface adhesion probably enables *A. hardii* to climb on an 80 deg incline but falls short of a critical threshold on vertical (90 deg) surfaces. Meanwhile, scansorial *Aneides* species and *P. glutinosus* have relatively larger feet that probably increase contact with the substrate to help them traverse vertical structures (Baken and O'Donnell, 2021; Aretz et al., 2022; Huie et al., 2025).

### Evidence for grasping ability in *Aneides*

Our findings support that scansorial species of *Aneides* can grasp broad surfaces. Grasping, as considered here, involves using the limbs to apply medially directed forces onto the substrate (lateral ground reaction forces) and generating enough friction to prevent the feet from slipping (Cartmill, 1974, 1985). Lizards and frogs accomplish the former by ‘hugging’ the surface by broadening the lateral spread of their limbs and pulling towards the midline (Autumn et al., 2006; Endlein et al., 2013). Indeed, we found that *A. aeneus* and *A. lugubris* laterally spread their long hindlimbs more than other salamanders. Lizards prevent their feet from slipping using their claws to grip the surface, while many frogs rely on their adhesive toe pads (Autumn et al., 2006; Endlein et al., 2013, 2017; Naylor and Higham, 2019). However, plethodontid salamanders lack these morphological structures. To avoid slipping, scansorial *Aneides* species may use a combination of their large feet for mucus adhesion and their digits to interlock with the substrate (Baken and Adams, 2019; O'Donnell and Deban, 2020b). Scansorial *Aneides* species also orient their feet more laterally on inclines, which is a better position to prevent slip in the mediolateral direction. Together, these behavioral adjustments suggest that the scansorial species of *Aneides* make the postural changes needed to grasp.

Scansorial species of *Aneides* possess multiple morphological modifications in their feet that likely aid grasping or, at the very least, enhance gripping ability. For instance, scansorial species of *Aneides* have modified phalangeal morphologies that should increase force production and grip strength compared with non-climbers (Huie et al., 2025). Arboreal A*. vagrans*, and presumably other species of *Aneides*, can also rapidly change the compliance of their sub-digital toe pads to increase foot–surface contact (Ritter and Miller, 1899; Brown et al., 2025; Raygoza and Staub, 2025). Compliant toe pads can deform around small surface asperities and increase friction on coarse surfaces (Langowski et al., 2018a,b). Finally, all species of *Aneides* except for *A. hardii* have unique carpal and tarsal arrangements with more articulating surfaces that purportedly increase spreading of the digits and improve force transmission (Wake, 1963, 1966). Digits that are more spread out would allow climbers to distribute forces across multiple directions, like many pad-bearing gecko species (Imburgia et al., 2019). Indeed, *A. aeneus* and *A. lugubris* spread the digits of their hindfeet further apart than do the other species and are largely unaffected by inclines (Fig. 7). This behavior, when coupled with lateral rotation of the feet, allows these species to simultaneously produce fore–aft forces for propulsion and medially directed forces for grasping. In contrast, *A. hardii* and *P. glutinous* actively reduce the digital spread of their hindfeet when climbing and align them with the direction of travel, limiting force transmission to the fore–aft direction (Zhuang and Higham, 2016). The patterns emerging from our study lay the groundwork to empirically confirm the direction and magnitude of the ground reaction forces that are generated during climbing and further examine the biomechanical strategies in salamanders that converge on or diverge from those of other scansorial tetrapods.

### Modulation of the limb function

In contrast to prior studies on lizards, we did not find kinematic evidence that the forelimbs take on a different functional role during climbing compared with walking. Salamander forelimb and hindlimb kinematics were overall modulated in similar ways in response to inclines. Meanwhile, lizards decouple the coordination of their forelimb and hindlimb movements during climbing (Birn-Jeffery and Higham, 2014; Foster and Higham, 2012; Zhuang and Higham, 2016), which reflects the forelimbs taking on a relatively larger propulsive role during climbing (Autumn et al., 2006; Wang et al., 2015). Differences in relative limb length may explain these discrepancies, as lizards have substantially longer hindlimbs than forelimbs (Foster et al., 2018; Huie et al., 2021) and our focal salamanders have fairly equal limb lengths (Huie et al., 2025).

While climbing salamanders do not appear to change the function of their limbs, they may increase the propulsive role of their axial system. During level walking, propulsion is generally determined 56–62% by femur retraction, 26–28% by long-axis rotation of the femur about the hip and 10–18% by rotation of the pelvic girdle (Edwards, 1977). We found that scansorial species of *Aneides* exhibit larger excursions of their pectoral and pelvic girdles during climbing compared with the less scansorial species. Maintaining increased pelvic girdle rotation with only slight increases in long-axis rotation and decreases in limb retraction may reflect a greater reliance on the axial system for generating propulsion on inclines, a hypothesis also proposed for some lizards (Jayne and Irschick, 1999). Wider excursions about the girdle also contribute to longer stride lengths that can reduce the energetic costs of locomotion (Wang et al., 2018), suggesting that scansorial species may be mitigating some of the higher costs of climbing through kinematic adjustments that improve the effectiveness of each step. Overall, our findings highlight a strong need to quantify climbing kinetics in salamanders to determine the functional roles of the forelimbs and hindlimbs with regards to grasping, clinging and propulsion.

### Other factors affect climbing ability

Allometry, tail use and microhabitat preference were not investigated in this study, but may broadly affect variation in climbing kinematics and ability. Variation in body size can affect locomotor performance and kinematics during level walking and has a substantially larger effect on climbing (Birn-Jeffery and Higham, 2016; Clemente et al., 2013; Labonte and Federle, 2015; Young et al., 2024b). Small salamanders have better clinging ability than large salamanders because of the higher surface area to volume ratio in the former (O'Donnell and Deban, 2020a). Thus, larger climbers must evolve larger or more effective morphological traits for substrate attachment (Labonte and Federle, 2015; Labonte et al., 2016) and make more deliberate kinematic adjustments (Birn-Jeffery and Higham, 2014). In this study, we held body size constant with our LMMs and compared EMMs assuming a constant body size. However, the body mass of our sampled individuals varied substantially from 0.4 to 9 g and the climbing kinematics of smaller salamanders may differ from those of larger individuals. Re-examination of these data may reveal that allometry affects climbing kinematics within and across salamander species. For instance, we noticed that smaller individuals often climbed with their venters off the substrate, consistent with lower requirements for maintaining adhesion and stability in smaller organisms.

Salamander tails vary in length and the degree of prehensility, but their functional roles remain relatively understudied (Dickie, 1999). Variation in tail use might impact climbing performance by affecting hindlimb kinematics. Some muscles connect the femur and pelvic girdle to the tail, including the relatively large caudofemoralis muscle, which plays an important role during limb retraction (Ashley-Ross, 1992). In general, tails often aid in stabilization during climbing and can be used as an auxiliary support structure (Stebbins, 1947; Jusufi et al., 2008; Luger et al., 2021; Dickinson et al., 2023; Young et al., 2024a). For instance, *Hydromantes platycephalus*, a montane climbing salamander, uses its prehensile tail like a walking stick when navigating steep inclines (Stebbins, 1947). While all species of *Aneides* have fairly prehensile tails (Dickie, 1999), we did not observe tail movements akin to those employed by *H. platycephalus*. Instead, we found that all of our study species drag their tails when walking but often bend them laterally to varying degrees when climbing. We suspect that bending of the tail increases leverage for stabilization purposes as well as reducing the energetic costs normally associated with overcoming the friction caused by dragging the tail (Willey et al., 2004). Investigating tail kinematics and its coordination with limb kinematics could provide additional insights into the functional role of the tail during climbing.

Furthermore, species may exhibit substrate-dependent variation in climbing performance and might climb faster on substrates found in their natural environments (Irschick et al., 2005; Wright et al., 2021). For example, surface texture can have profound consequences on climbing performance because rough surfaces disrupt mucosal adhesion (O'Donnell and Deban, 2020b). Coarse surfaces with intermediate roughness (asperity size: 100–350 μm), similar to our experimental substrate (250–540 μm), are associated with low clinging performance (O'Donnell and Deban, 2020b). Meanwhile, on substrates with large irregularities (>1000 μm), salamanders can mechanically interlock their digits and tail with the surface to secure their attachment. Our finding that *A. aeneus* climbs faster than *A. lugubris* may be attributable, in part, to the experimental substrate being more similar to the coarse rock faces that *A. aeneus* regularly climbs rather than the rugose tree bark that *A. lugubris* associates with. Arboreal *A. vagrans* studied by Aretz et al. (2022) climbed a rough vertical surface about 1.65 times faster than the *A. aeneus* in this study, underscoring the effects of habitat preference and surface texture on climbing performance. However, maximal speeds were encouraged from *A. vagrans*, while we studied volitional climbing speeds, so it is difficult to make direct comparisons. Nevertheless, future studies should investigate the impact of substrate properties (i.e. texture, width and compliance) on walking and climbing performance to assess whether scansorial species experience increased performance on preferred substrates.

Finally, some of the kinematic changes that we observed on steeper inclines may be associated with changes in speed rather than a direct response to incline angle. As animals increase or decrease their locomotor speed, they often change their gait and limb kinematics (Hoyt and Taylor, 1981). As salamanders naturally decrease their locomotor speed on steeper inclines, it is difficult to discern which changes in limb kinematics are associated with altering speed. Changes in foot angle and limb spread are likely direct responses to the incline as mechanisms to improve attachment, but it is less clear for spatiotemporal gait variables, as locomotor speed can be regulated by changes in stride length, stride frequency or both (Hanna et al., 2022; Ekhator et al., 2023). Thus, future studies should consider collecting additional data to investigate whether inclines affect stride length or frequency when assuming a constant speed. This could be accomplished by statistically analyzing kinematic and spatiotemporal gait traits throughout a restricted range of locomotor speeds employed by the animal across all inclines. Additionally, the use of physical robotic models (Karakasiliotis et al., 2013) or digital 3D models (Kawano et al., 2024) could enable researchers to explore kinematic changes across a wider range of speeds and help disentangle the relationships between locomotor kinematics, speed and incline.

### Implications for the evolution of climbing

Comparisons of closely related salamander species that span a gradient from ground dwelling to highly scansorial provide insights into evolutionary transitions towards scansorial habitats. Species that lack specific morphologies for improving climbing performance are still capable of climbing steep inclines (i.e. *A. hardii*, *P. glutinosus* and *P. cinereus*), demonstrating that specialized climbing morphologies are not required to be scansorial. Therefore, the capacity to climb through kinematic adjustments probably precedes the evolution of morphological adaptations that improve climbing performance. This is crucial for interpreting evolutionary transitions to scansoriality from ancestral states that experience selective pressures that conflict with climbing pressures. For instance, fossoriality promotes short, diminutive limbs among plethodontid salamanders (Wake, 1991). Yet, arboreal and fossorial lineages have repeatedly evolved from ancestors with locomotor morphologies that resemble terrestrial lineages with moderate limb lengths (Parra-Olea and Wake, 2001). To this end, behavior can mediate the relationship between morphology and performance, allowing the hundreds of plethodontid species that lack climbing phenotypes or possess an entirely separate suite of traits (i.e. the webbed feet of *Bolitoglossa*) to access scansorial habitats. Morphological adaptations for climbing are likely to evolve in lineages once climbing becomes habitual and the benefits of enhanced climbing performance outweigh potential trade-offs. Such a scenario may explain the process by which some *Aneides* species evolved strong climbing tendencies and their presumed morphological adaptations. In conclusion, integrating information about behavior, morphology and performance can provide insights into the widespread scansorial tendencies of plethodontid salamanders and evolutionary origins of habitat transitions.

## Supplementary Material

10.1242/jexbio.251894_sup1 Supplementary information
