# Supplementary material for: Limb kinematics and morphology improve salamander climbing performance
Source: J Exp Biol. 2026 Jun 4;229(11):jeb251894. doi: 10.1242/jeb.251894 (PMC13286359; doi:10.1242/jeb.251894)
Supplement: Supplementary information [file jexbio-229-251894-s1.pdf]

Table S1. Results for the fixed effects that explain significant variation in spatiotemporal gait parameters and kinematics. F-tests were performed on linear mixed effect models with the model structure: var ~ Incline * Species * Limb + log(SVL) + (1|ID). Factors that were **not** statistically significant (p > 0.05) are marked in bold. R^2_{cond} represents the variance explained by the fixed and random effects; R^2_{marg} represents the variance explained by only the fixed effects. Sample sizes for each treatment are in Table 1.

Variable	Fixed Effect	numDF	denDF	F-value	p-value	R^2_{cond}/R^2_{marg}
Speed (SVL/s)	(Intercept)	1	682	375.24	< 0.001	0.756/0.607
	Incline	3	682	373.29	< 0.001	
	Species	3	36	17.06	< 0.001	
	log(SVL)	1	682	14.30	< 0.001	
	Incline*Species	9	682	8.89	< 0.001	
Duty Factor	(Intercept)	1	1401	81168.40	< 0.001	0.770/0.648
	Incline	3	1401	1082.52	< 0.001	
	Species	3	36	1.66	**0.194**	
	Limb	1	1401	389.84	< 0.001	
	log(SVL)	1	1401	13.05	0.001	
	Incline*Species	9	1401	13.55	< 0.001	
	Incline*Limb	3	1401	17.97	< 0.001	
	Species*Limb	3	1401	12.26	< 0.001	
	Incline*Species*Limb	9	1401	2.54	0.007	
Stride Length (SVL)	(Intercept)	1	1401	4922.22	< 0.001	0.846/0.683
	Incline	3	1401	992.60	< 0.001	
	Species	3	36	32.90	< 0.001	
	Limb	1	1401	2.12	**0.145**	
	log(SVL)	1	1401	17.68	0.000	
	Incline*Species	9	1401	15.20	< 0.001	
	Incline*Limb	3	1401	2.09	**0.099**	
	Species*Limb	3	1401	0.16	**0.924**	
	Incline*Species*Limb	9	1401	0.23	**0.991**	
Stride Frequency (strides/s)	(Intercept)	1	1401	576.75	< 0.001	0.766/0.616
	Incline	3	1401	815.84	< 0.001	
	Species	3	36	16.22	< 0.001	
	Limb	1	1401	0.08	**0.772**	
	log(SVL)	1	1401	13.36	< 0.001	
	Incline*Species	9	1401	19.98	< 0.001	
	Incline*Limb	3	1401	0.03	**0.992**	
	Species*Limb	3	1401	0.02	**0.996**	
	Incline*Species*Limb	9	1401	0.06	**1.000**	
Max Abduction/Adduction (°)	(Intercept)	1	1401	11440.92	< 0.001	0.572/0.460
	Incline	3	1401	36.07	< 0.001	
	Species	3	36	30.36	< 0.001	
	Limb	1	1401	172.71	< 0.001	
	log(SVL)	1	1401	3.93	0.048	
	Incline*Species	9	1401	4.01	< 0.001	
	Incline*Limb	3	1401	28.23	< 0.001	
	Species*Limb	3	1401	49.07	< 0.001	
	Incline*Species*Limb	9	1401	5.65	< 0.001	
Min Abduction/Adduction (°)	(Intercept)	1	1401	13.56	< 0.001	0.590/0.509
	Incline	3	1401	104.01	< 0.001	
	Species	3	36	38.45	< 0.001	
	Limb	1	1401	504.36	< 0.001	
	log(SVL)	1	1401	9.62	0.002	
	Incline*Species	9	1401	10.57	< 0.001	

Journal of Experimental Biology • Supplementary information

	Incline*Limb	3	1401	0.69	**0.558**	
	Species*Limb	3	1401	9.02	< 0.001	
	Incline*Species*Limb	9	1401	0.68	**0.729**	
Abduction/Adduction Excursion (°)	(Intercept)	1	1401	9092.43	< 0.001	0.565/0.456
	Incline	3	1401	7.14	< 0.001	
	Species	3	36	11.43	< 0.001	
	Limb	1	1401	686.41	< 0.001	
	log(SVL)	1	1401	12.75	< 0.001	
	Incline*Species	9	1401	2.97	0.002	
	Incline*Limb	3	1401	24.07	< 0.001	
	Species*Limb	3	1401	57.26	< 0.001	
	Incline*Species*Limb	9	1401	5.09	< 0.001	
Max Protraction/Retraction (°)	(Intercept)	1	1401	4576.64	< 0.001	0.758/0.664
	Incline	3	1401	100.32	< 0.001	
	Species	3	36	12.59	< 0.001	
	Limb	1	1401	2620.14	< 0.001	
	log(SVL)	1	1401	6.61	0.010	
	Incline*Species	9	1401	13.04	< 0.001	
	Incline*Limb	3	1401	16.20	< 0.001	
	Species*Limb	3	1401	115.59	< 0.001	
	Incline*Species*Limb	9	1401	3.53	< 0.001	
Min Protraction/Retraction (°)	(Intercept)	1	1401	12551.87	< 0.001	0.550/0.427
	Incline	3	1401	52.90	< 0.001	
	Species	3	36	20.17	< 0.001	
	Limb	1	1401	7.66	0.006	
	log(SVL)	1	1401	0.08	**0.782**	
	Incline*Species	9	1401	2.28	0.015	
	Incline*Limb	3	1401	9.96	< 0.001	
	Species*Limb	3	1401	171.93	< 0.001	
	Incline*Species*Limb	9	1401	2.80	0.003	
Protraction/Retraction Excursion (°)	(Intercept)	1	1401	20915.75	< 0.001	0.704/0.627
	Incline	3	1401	62.02	< 0.001	
	Species	3	36	23.89	< 0.001	
	Limb	1	1401	1395.20	< 0.001	
	log(SVL)	1	1401	4.62	0.032	
	Incline*Species	9	1401	9.29	< 0.001	
	Incline*Limb	3	1401	27.09	< 0.001	
	Species*Limb	3	1401	160.29	< 0.001	
	Incline*Species*Limb	9	1401	2.07	0.029	
Max Long-Axis Rotation (°)	(Intercept)	1	1401	4920.72	< 0.001	0.916/0.889
	Incline	3	1401	36.75	< 0.001	
	Species	3	36	4.14	0.013	
	Limb	1	1401	14380.13	< 0.001	
	log(SVL)	1	1401	2.01	**0.157**	
	Incline*Species	9	1401	3.81	< 0.001	
	Incline*Limb	3	1401	93.98	< 0.001	
	Species*Limb	3	1401	183.44	< 0.001	
	Incline*Species*Limb	9	1401	2.93	0.002	
Min Long-Axis Rotation (°)	(Intercept)	1	1401	4130.40	< 0.001	0.994/0.935
	Incline	3	1401	5.05	0.002	
	Species	3	36	15.30	< 0.001	
	Limb	1	1401	23558.47	< 0.001	
	log(SVL)	1	1401	8.48	0.004	
	Incline*Species	9	1401	5.20	< 0.001	

	Incline*Limb	3	1401	7.24	< 0.001	
	Species*Limb	3	1401	111.63	< 0.001	
	Incline*Species*Limb	9	1401	3.44	< 0.001	
Long-Axis Rotation Excursion (°)	(Intercept)	1	1401	10926.41	< 0.001	0.622/0.553
	Incline	3	1401	21.49	< 0.001	
	Species	3	36	16.82	< 0.001	
	Limb	1	1401	1465.53	< 0.001	
	log(SVL)	1	1401	0.86	**0.354**	
	Incline*Species	9	1401	3.65	< 0.001	
	Incline*Limb	3	1401	24.97	< 0.001	
	Species*Limb	3	1401	46.66	< 0.001	
	Incline*Species*Limb	9	1401	1.85	**0.056**	
Max Elbow/Knee (°)	(Intercept)	1	1401	97706.14	< 0.001	0.746/0.681
	Incline	3	1401	15.14	< 0.001	
	Species	3	36	17.10	< 0.001	
	Limb	1	1401	3139.49	< 0.001	
	log(SVL)	1	1401	1.10	**0.295**	
	Incline*Species	9	1401	6.64	< 0.001	
	Incline*Limb	3	1401	1.03	**0.377**	
	Species*Limb	3	1401	47.46	< 0.001	
	Incline*Species*Limb	9	1401	2.83	0.003	
Min Elbow/Knee (°)	(Intercept)	1	1401	7355.67	< 0.001	0.461/0.287
	Incline	3	1401	55.12	< 0.001	
	Species	3	36	6.91	< 0.001	
	Limb	1	1401	0.04	**0.850**	
	log(SVL)	1	1401	5.45	0.020	
	Incline*Species	9	1401	3.42	< 0.001	
	Incline*Limb	3	1401	16.01	< 0.001	
	Species*Limb	3	1401	53.83	< 0.001	
	Incline*Species*Limb	9	1401	1.88	**0.052**	
Elbow/Knee Excursion (°)	(Intercept)	1	1401	7951.58	< 0.001	0.594/0.487
	Incline	3	1401	75.46	< 0.001	
	Species	3	36	7.85	< 0.001	
	Limb	1	1401	1043.98	< 0.001	
	log(SVL)	1	1401	9.08	0.003	
	Incline*Species	9	1401	3.47	< 0.001	
	Incline*Limb	3	1401	14.21	< 0.001	
	Species*Limb	3	1401	13.21	< 0.001	
	Incline*Species*Limb	9	1401	2.19	0.021	
Max Wrist/Ankle (°)	(Intercept)	1	1401	318725.90	< 0.001	0.530/0.442
	Incline	3	1401	74.00	< 0.001	
	Species	3	36	16.30	< 0.001	
	Limb	1	1401	538.80	< 0.001	
	log(SVL)	1	1401	0.20	**0.685**	
	Incline*Species	9	1401	7.80	< 0.001	
	Incline*Limb	3	1401	7.10	< 0.001	
	Species*Limb	3	1401	56.30	< 0.001	
	Incline*Species*Limb	9	1401	4.30	< 0.001	
Min Wrist/Ankle (°)	(Intercept)	1	1401	13207.63	< 0.001	0.508/0.442
	Incline	3	1401	5.75	< 0.001	
	Species	3	36	8.69	< 0.001	
	Limb	1	1401	1058.71	< 0.001	
	log(SVL)	1	1401	0.15	**0.697**	
	Incline*Species	9	1401	3.59	< 0.001	

Journal of Experimental Biology • Supplementary information

	Incline*Limb	3	1401	2.23	**0.084**	
	Species*Limb	3	1401	17.07	< 0.001	
	Incline*Species*Limb	9	1401	0.91	**0.515**	
Wrist/Ankle Excursion (°)	(Intercept)	1	1401	12740.67	< 0.001	0.416/0.323
	Incline	3	1401	14.52	< 0.001	
	Species	3	36	11.17	< 0.001	
	Limb	1	1401	525.02	< 0.001	
	log(SVL)	1	1401	0.04	**0.834**	
	Incline*Species	9	1401	3.50	< 0.001	
	Incline*Limb	3	1401	1.38	**0.249**	
	Species*Limb	3	1401	3.27	0.021	
	Incline*Species*Limb	9	1401	1.57	**0.118**	
Max Contralateral Yaw (°)	(Intercept)	1	1401	5100.71	< 0.001	0.467/0.418
	Incline	3	1401	40.48	< 0.001	
	Species	3	36	65.14	< 0.001	
	Limb	1	1401	9.86	0.002	
	log(SVL)	1	1401	0.53	**0.467**	
	Incline*Species	9	1401	4.26	< 0.001	
	Incline*Limb	3	1401	30.04	< 0.001	
	Species*Limb	3	1401	34.83	< 0.001	
	Incline*Species*Limb	9	1401	5.36	< 0.001	
Max Ipsilateral Yaw (°)	(Intercept)	1	1401	2832.51	< 0.001	0.465/0.355
	Incline	3	1401	20.62	< 0.001	
	Species	3	36	29.07	< 0.001	
	Limb	1	1401	25.03	< 0.001	
	log(SVL)	1	1401	13.53	< 0.001	
	Incline*Species	9	1401	2.86	0.002	
	Incline*Limb	3	1401	14.76	< 0.001	
	Species*Limb	3	1401	14.29	< 0.001	
	Incline*Species*Limb	9	1401	2.07	0.029	
Yaw Excursion (°)	(Intercept)	1	1401	5356.77	< 0.001	0.599/0.516
	Incline	3	1401	54.84	< 0.001	
	Species	3	36	60.51	< 0.001	
	Limb	1	1401	33.00	< 0.001	
	log(SVL)	1	1401	9.52	0.002	
	Incline*Species	9	1401	6.18	< 0.001	
	Incline*Limb	3	1401	39.61	< 0.001	
	Species*Limb	3	1401	28.01	< 0.001	
	Incline*Species*Limb	9	1401	5.30	< 0.001	
Foot Angle (°)	(Intercept)	1	1401	46.73	< 0.001	0.580/0.481
	Incline	3	1401	183.50	< 0.001	
	Species	3	36	17.06	< 0.001	
	Limb	1	1401	117.77	< 0.001	
	log(SVL)	1	1401	3.61	**0.058**	
	Incline*Species	9	1401	8.64	< 0.001	
	Incline*Limb	3	1401	23.70	< 0.001	
	Species*Limb	3	1401	56.93	< 0.001	
	Incline*Species*Limb	9	1401	5.71	< 0.001	
Digital Spread (°)	(Intercept)	1	1401	24393.99	< 0.001	0.697/0.513
	Incline	3	1401	131.88	< 0.001	
	Species	3	36	6.35	0.001	
	Limb	1	1401	1319.29	< 0.001	
	log(SVL)	1	1401	3.19	**0.074**	
	Incline*Species	9	1401	13.51	< 0.001	

Journal of Experimental Biology • Supplementary information

	Incline*Limb	3	1401	1.41	**0.238**	
	Species*Limb	3	1401	72.02	< 0.001	
	Incline*Species*Limb	9	1401	3.50	< 0.001	
Limb Spread (SVL)	(Intercept)	1	1401	6854.13	< 0.001	0.672/0.592
	Incline	3	1401	14.85	< 0.001	
	Species	3	36	30.40	< 0.001	
	Limb	1	1401	1161.34	< 0.001	
	log(SVL)	1	1401	20.27	< 0.001	
	Incline*Species	9	1401	2.95	0.002	
	Incline*Limb	3	1401	6.45	< 0.001	
	Species*Limb	3	1401	66.49	< 0.001	
	Incline*Species*Limb	9	1401	4.68	< 0.001	
Max Hip Height (SVL)	(Intercept)	1	1401	1795.48	< 0.001	0.668/0.471
	Incline	3	1401	283.52	< 0.001	
	Species	3	36	11.49	< 0.001	
	Limb	1	1401	173.39	< 0.001	
	log(SVL)	1	1401	15.57	< 0.001	
	Incline*Species	9	1401	9.29	< 0.001	
	Incline*Limb	3	1401	2.61	**0.050**	
	Species*Limb	3	1401	1.14	**0.332**	
	Incline*Species*Limb	9	1401	4.96	< 0.001	
Min Hip Height (SVL)	(Intercept)	1	1401	871.51	< 0.001	0.548/0.310
	Incline	3	1401	142.90	< 0.001	
	Species	3	36	5.25	0.004	
	Limb	1	1401	54.33	< 0.001	
	log(SVL)	1	1401	9.99	0.002	
	Incline*Species	9	1401	9.30	< 0.001	
	Incline*Limb	3	1401	1.15	**0.329**	
	Species*Limb	3	1401	4.44	0.004	
	Incline*Species*Limb	9	1401	2.08	0.028	
Hip Height Excursion (SVL)	(Intercept)	1	1401	2081.08	< 0.001	0.638/0.554
	Incline	3	1401	111.46	< 0.001	
	Species	3	36	14.49	< 0.001	
	Limb	1	1401	1229.85	< 0.001	
	log(SVL)	1	1401	10.78	0.001	
	Incline*Species	9	1401	6.18	< 0.001	
	Incline*Limb	3	1401	6.18	< 0.001	
	Species*Limb	3	1401	16.56	< 0.001	
	Incline*Species*Limb	9	1401	12.59	< 0.001	

Table S2. Comparisons of mean values for the spatiotemporal gait parameters and limb kinematics. Linear mixed-effects models for each variable were performed with the model structure: var ~ Incline * Species * Limb + log(SVL) + (1|ID), except for speed which did not include a limb as a fixed effect. Estimated marginal means of the fixed effects (EMMs), standard error (se), and 95% confidence intervals (CI) were estimated assuming a constant SVL of 4.94 cm. Sample sizes for each treatment are reported in Table 1.

			Aneides aeneus		*Aneides lugubris*		*Aneides hardii*		*Plethodon glutinosus*	
**Variable**	**Limb**	**Incline**	**EMM ± se**	**CI**	**EMM ± se**	**CI**	**EMM ± se**	**CI**	**EMM ± se**	**CI**
Speed (SVL/s)	-	0°	1.62 ± 0.08	1.45, 1.78	1.09 ± 0.08	0.57, 0.89	0.73 ± 0.08	0.93, 1.25	0.93 ± 0.08	0.76, 1.09
	-	45°	1.35 ± 0.08	1.19, 1.51	0.81 ± 0.08	0.37, 0.69	0.53 ± 0.08	0.65, 0.97	0.82 ± 0.08	0.66, 0.98
	-	80°	0.63 ± 0.08	0.46, 0.79	0.47 ± 0.08	-0.05, 0.28	0.11 ± 0.08	0.31, 0.64	0.34 ± 0.08	0.18, 0.50
	-	90°	0.40 ± 0.08	0.24, 0.56	0.30 ± 0.08	-0.22, 0.51	0.15 ± 0.18	0.13, 0.47	0.22 ± 0.08	0.06, 0.38
Duty Factor	Fore	0°	0.82 ± 0.01	0.81, 0.83	0.81 ± 0.01	0.80, 0.83	0.82 ± 0.01	0.80, 0.82	0.82 ± 0.01	0.80, 0.83
	Fore	45°	0.85 ± 0.01	0.83, 0.86	0.83 ± 0.01	0.83, 0.86	0.84 ± 0.01	0.82, 0.85	0.81 ± 0.01	0.80, 0.83
	Fore	80°	0.90 ± 0.01	0.88, 0.91	0.89 ± 0.01	0.93, 0.96	0.94 ± 0.01	0.87, 0.90	0.89 ± 0.01	0.88, 0.90
	Fore	90°	0.91 ± 0.01	0.90, 0.93	0.92 ± 0.01	0.92, 0.98	0.95 ± 0.02	0.90, 0.93	0.92 ± 0.01	0.91, 0.94
	Hind	0°	0.85 ± 0.01	0.84, 0.87	0.84 ± 0.01	0.84, 0.87	0.85 ± 0.01	0.83, 0.86	0.85 ± 0.01	0.84, 0.86
	Hind	45°	0.87 ± 0.01	0.86, 0.88	0.87 ± 0.01	0.86, 0.89	0.87 ± 0.01	0.86, 0.89	0.88 ± 0.01	0.86, 0.89
	Hind	80°	0.92 ± 0.01	0.90, 0.93	0.90 ± 0.01	0.93, 0.95	0.94 ± 0.01	0.89, 0.91	0.92 ± 0.01	0.91, 0.94
	Hind	90°	0.93 ± 0.01	0.91, 0.94	0.92 ± 0.01	0.93, 0.99	0.96 ± 0.02	0.91, 0.94	0.95 ± 0.01	0.93, 0.96
Stride Length (SVL)	Fore	0°	0.62 ± 0.02	0.59, 0.66	0.68 ± 0.02	0.44, 0.51	0.47 ± 0.02	0.65, 0.71	0.62 ± 0.02	0.58, 0.65
	Fore	45°	0.62 ± 0.02	0.58, 0.65	0.64 ± 0.02	0.44, 0.51	0.47 ± 0.02	0.61, 0.67	0.60 ± 0.02	0.57, 0.64
	Fore	80°	0.54 ± 0.02	0.50, 0.57	0.56 ± 0.02	0.29, 0.36	0.32 ± 0.02	0.52, 0.59	0.47 ± 0.02	0.44, 0.50
	Fore	90°	0.50 ± 0.02	0.46, 0.53	0.49 ± 0.02	0.27, 0.39	0.33 ± 0.03	0.45, 0.52	0.42 ± 0.02	0.39, 0.46
	Hind	0°	0.63 ± 0.02	0.59, 0.66	0.67 ± 0.02	0.44, 0.51	0.47 ± 0.02	0.64, 0.71	0.61 ± 0.02	0.58, 0.64
	Hind	45°	0.62 ± 0.02	0.58, 0.65	0.64 ± 0.02	0.44, 0.50	0.47 ± 0.02	0.61, 0.67	0.60 ± 0.02	0.56, 0.63
	Hind	80°	0.55 ± 0.02	0.52, 0.58	0.57 ± 0.02	0.31, 0.37	0.34 ± 0.02	0.53, 0.60	0.49 ± 0.02	0.45, 0.52
	Hind	90°	0.50 ± 0.02	0.47, 0.53	0.49 ± 0.02	0.24, 0.37	0.30 ± 0.03	0.46, 0.53	0.43 ± 0.02	0.40, 0.46
Stride Frequency (stride/s)	Fore	0°	2.50 ± 0.11	2.27, 2.73	1.56 ± 0.11	1.28, 1.73	1.51 ± 0.11	1.33, 1.78	1.51 ± 0.11	1.28, 1.73
	Fore	45°	2.11 ± 0.11	1.88, 2.34	1.23 ± 0.11	0.86, 1.31	1.08 ± 0.11	1.01, 1.46	1.33 ± 0.11	1.10, 1.55
	Fore	80°	1.13 ± 0.11	0.90, 1.35	0.81 ± 0.11	0.13, 0.58	0.35 ± 0.11	0.58, 1.04	0.66 ± 0.11	0.44, 0.88
	Fore	90°	0.80 ± 0.11	0.58, 1.03	0.61 ± 0.12	-0.08, 0.91	0.42 ± 0.25	0.37, 0.85	0.47 ± 0.11	0.24, 0.69
	Hind	0°	2.52 ± 0.11	2.30, 2.75	1.56 ± 0.11	1.25, 1.71	1.48 ± 0.11	1.33, 1.78	1.47 ± 0.11	1.25, 1.70
	Hind	45°	2.12 ± 0.11	1.89, 2.35	1.23 ± 0.11	0.84, 1.29	1.06 ± 0.11	1.00, 1.45	1.33 ± 0.11	1.11, 1.56
	Hind	80°	1.09 ± 0.11	0.86, 1.32	0.80 ± 0.11	0.13, 0.58	0.35 ± 0.11	0.57, 1.03	0.66 ± 0.11	0.43, 0.88
	Hind	90°	0.81 ± 0.11	0.58, 1.04	0.61 ± 0.12	-0.09, 0.91	0.41 ± 0.25	0.37, 0.84	0.47 ± 0.11	0.25, 0.69

Journal of Experimental Biology • Supplementary information

Max Abduction/ Adduction (°)	Fore	0°	35.36 ± 1.03	33.27, 37.45	38.49 ± 1.02	49.33, 53.49	51.41 ± 1.02	36.41, 40.57	42.31 ± 1.02	40.23, 44.38
	Fore	45°	37.52 ± 1.03	35.42, 39.61	41.51 ± 1.02	48.45, 52.61	50.53 ± 1.02	39.43, 43.59	47.14 ± 1.02	45.06, 49.21
	Fore	80°	42.56 ± 1.03	40.47, 44.66	43.37 ± 1.05	49.36, 53.51	51.43 ± 1.02	41.25, 45.49	51.97 ± 1.02	49.90, 54.05
	Fore	90°	42.37 ± 1.03	40.27, 44.46	43.99 ± 1.12	41.40, 53.18	47.29 ± 2.90	41.72, 46.25	49.53 ± 1.02	47.46, 51.61
	Hind	0°	35.58 ± 1.03	33.49, 37.67	41.64 ± 1.02	41.01, 45.17	43.09 ± 1.02	39.57, 43.72	42.81 ± 1.02	40.74, 44.89
	Hind	45°	35.86 ± 1.03	33.77, 37.95	44.24 ± 1.02	45.70, 49.86	47.78 ± 1.02	42.17, 46.32	43.64 ± 1.02	41.57, 45.72
	Hind	80°	36.39 ± 1.03	34.30, 38.48	44.43 ± 1.05	42.71, 46.87	44.79 ± 1.02	42.31, 46.55	43.26 ± 1.02	41.18, 45.33
	Hind	90°	34.37 ± 1.03	32.28, 36.46	43.01 ± 1.12	37.34, 49.12	43.23 ± 2.90	40.74, 45.27	40.72 ± 1.02	38.65, 42.80
Min Abduction/ Adduction (°)	Fore	0°	-8.94 ± 0.75	-10.46, -7.42	-7.91 ± 0.75	-0.28, 2.74	1.23 ± 0.75	-9.42, -6.40	-8.10 ± 0.74	-9.61, -6.59
	Fore	45°	-3.72 ± 0.75	-5.24, -2.20	-4.83 ± 0.75	0.94, 3.96	2.45 ± 0.75	-6.34, -3.32	-3.35 ± 0.74	-4.86, -1.84
	Fore	80°	-2.96 ± 0.75	-4.48, -1.44	-4.17 ± 0.76	0.30, 3.32	1.81 ± 0.75	-5.72, -2.62	-2.22 ± 0.74	-3.73, -0.71
	Fore	90°	-2.28 ± 0.75	-3.80, -0.76	-5.03 ± 0.82	-3.95, 5.20	0.62 ± 2.25	-6.70, -3.36	-2.88 ± 0.74	-4.39, -1.37
	Hind	0°	-2.79 ± 0.75	-4.31, -1.27	-5.15 ± 0.75	4.27, 7.29	5.78 ± 0.75	-6.66, -3.64	-3.93 ± 0.74	-5.44, -2.42
	Hind	45°	0.89 ± 0.75	-0.63, 2.41	-2.31 ± 0.75	4.56, 7.59	6.07 ± 0.75	-3.83, -0.80	2.04 ± 0.74	0.53, 3.55
	Hind	80°	2.66 ± 0.75	1.15, 4.18	-1.74 ± 0.76	4.63, 7.66	6.15 ± 0.75	-3.29, -0.19	2.45 ± 0.74	0.94, 3.96
	Hind	90°	2.93 ± 0.75	1.41, 4.45	-1.56 ± 0.82	-2.16, 6.98	2.41 ± 2.25	-3.23, 0.11	2.83 ± 0.74	1.32, 4.34
Abduction/ Addduction Excursion (°)	Fore	0°	44.31 ± 1.20	41.89, 46.73	46.40 ± 1.19	47.77, 52.58	50.18 ± 1.19	43.99, 48.80	50.41 ± 1.18	48.01, 52.81
	Fore	45°	41.24 ± 1.20	38.82, 43.66	46.33 ± 1.19	45.68, 50.49	48.09 ± 1.19	43.93, 48.74	50.49 ± 1.18	48.08, 52.89
	Fore	80°	45.53 ± 1.20	43.11, 47.95	47.55 ± 1.21	47.22, 52.03	49.62 ± 1.19	45.09, 50.00	54.19 ± 1.18	51.79, 56.59
	Fore	90°	44.65 ± 1.20	42.23, 47.07	49.03 ± 1.30	39.73, 53.53	46.63 ± 3.40	46.40, 51.66	52.41 ± 1.18	50.01, 54.81
	Hind	0°	38.37 ± 1.20	35.95, 40.79	46.79 ± 1.19	34.91, 39.72	37.31 ± 1.19	44.39, 49.20	46.74 ± 1.18	44.34, 49.14
	Hind	45°	34.98 ± 1.20	32.56, 37.40	46.56 ± 1.19	39.30, 44.11	41.70 ± 1.19	44.15, 48.96	41.60 ± 1.18	39.20, 44.00
	Hind	80°	33.73 ± 1.20	31.31, 36.15	46.18 ± 1.21	36.24, 41.05	38.64 ± 1.19	43.72, 48.63	40.80 ± 1.18	38.40, 43.20
	Hind	90°	31.45 ± 1.20	29.03, 33.87	44.58 ± 1.30	33.89, 47.69	40.79 ± 3.40	41.95, 47.21	37.90 ± 1.18	35.49, 40.30
Max Protraction/ Retraction (°)	Fore	0°	27.92 ± 1.46	24.97, 30.86	31.02 ± 1.44	33.34, 39.19	36.26 ± 1.44	28.10, 33.95	24.65 ± 1.44	21.73, 27.57
	Fore	45°	25.32 ± 1.46	22.37, 28.27	32.38 ± 1.44	34.35, 40.20	37.28 ± 1.44	29.45, 35.30	27.01 ± 1.44	24.09, 29.93
	Fore	80°	34.10 ± 1.46	31.16, 37.05	37.08 ± 1.47	36.89, 42.74	39.82 ± 1.44	34.11, 40.05	33.83 ± 1.44	30.91, 36.75
	Fore	90°	34.13 ± 1.46	31.19, 37.08	37.74 ± 1.55	33.31, 48.26	40.79 ± 3.68	34.60, 40.87	36.95 ± 1.44	34.03, 39.87
	Hind	0°	44.06 ± 1.46	41.11, 47.01	40.08 ± 1.44	47.13, 52.98	50.05 ± 1.44	37.15, 43.00	46.04 ± 1.44	43.12, 48.96
	Hind	45°	43.13 ± 1.46	40.18, 46.08	42.10 ± 1.44	54.43, 60.28	57.35 ± 1.44	39.18, 45.03	56.44 ± 1.44	53.52, 59.36
	Hind	80°	45.73 ± 1.46	42.78, 48.67	45.69 ± 1.47	50.14, 55.99	53.06 ± 1.44	42.72, 48.66	58.44 ± 1.44	55.52, 61.36
	Hind	90°	43.01 ± 1.46	40.07, 45.96	46.04 ± 1.55	55.16, 70.11	62.63 ± 3.68	42.90, 49.17	58.66 ± 1.44	55.74, 61.58
Min Protraction/ Retraction (°)	Fore	0°	-55.25 ± 1.22	-57.71, -52.78	-52.06 ± 1.21	-55.85, -50.95	-53.40 ± 1.21	-54.51, -49.61	-57.16 ± 1.21	-59.61, -54.72
	Fore	45°	-55.42 ± 1.22	-57.88, -52.95	-50.38 ± 1.21	-58.63, -53.73	-56.18 ± 1.21	-52.83, -47.93	-58.33 ± 1.21	-60.78, -55.89
	Fore	80°	-54.46 ± 1.22	-56.92, -51.99	-50.85 ± 1.23	-57.58, -52.68	-55.13 ± 1.21	-53.35, -48.35	-59.09 ± 1.21	-61.54, -56.64

Journal of Experimental Biology • Supplementary information

	Fore	90°	-51.43 ± 1.22	-53.89, -48.96	-47.99 ± 1.32	-56.54, -42.76	-49.65 ± 3.40	-50.66, -45.32	-56.30 ± 1.21	-58.74, -53.85
	Hind	0°	-45.96 ± 1.22	-48.43, -43.50	-56.71 ± 1.21	-46.53, -41.62	-44.07 ± 1.21	-59.16, -54.26	-61.19 ± 1.21	-63.64, -58.74
	Hind	45°	-48.60 ± 1.22	-51.06, -46.13	-60.81 ± 1.21	-53.16, -48.26	-50.71 ± 1.21	-63.26, -58.36	-66.69 ± 1.21	-69.13, -64.24
	Hind	80°	-47.60 ± 1.22	-50.07, -45.14	-59.86 ± 1.23	-50.51, -45.61	-48.06 ± 1.21	-62.36, -57.36	-60.93 ± 1.21	-63.38, -58.49
	Hind	90°	-44.51 ± 1.22	-46.98, -42.05	-52.86 ± 1.32	-45.02, -31.24	-38.13 ± 3.40	-55.53, -50.19	-55.85 ± 1.21	-58.30, -53.41
Protraction/ Retraction Excursion (°)	Fore	0°	83.16 ± 1.67	79.78, 86.55	83.08 ± 1.66	86.30, 93.03	89.66 ± 1.66	79.72, 86.45	81.81 ± 1.66	78.45, 85.17
	Fore	45°	80.74 ± 1.67	77.35, 84.13	82.76 ± 1.66	90.10, 96.83	93.46 ± 1.66	79.39, 86.12	85.34 ± 1.66	81.98, 88.70
	Fore	80°	88.56 ± 1.67	85.18, 91.95	87.91 ± 1.69	91.58, 98.31	94.95 ± 1.66	84.47, 91.34	92.92 ± 1.66	89.56, 96.28
	Fore	90°	85.56 ± 1.67	82.18, 88.95	85.72 ± 1.81	80.86, 99.96	90.41 ± 4.71	82.05, 89.39	93.24 ± 1.66	89.88, 96.60
	Hind	0°	90.03 ± 1.67	86.64, 93.41	96.79 ± 1.66	90.76, 97.49	94.13 ± 1.66	93.42, 100.15	107.22 ± 1.66	103.86, 110.58
	Hind	45°	91.73 ± 1.67	88.34, 95.12	102.92 ± 1.66	104.70, 111.43	108.07 ± 1.66	99.55, 106.28	123.13 ± 1.66	119.77, 126.49
	Hind	80°	93.33 ± 1.67	89.95, 96.72	105.52 ± 1.69	97.76, 104.49	101.13 ± 1.66	102.09, 108.96	119.37 ± 1.66	116.01, 122.73
	Hind	90°	87.53 ± 1.67	84.14, 90.92	98.89 ± 1.81	91.18, 110.28	100.73 ± 4.71	95.22, 102.56	114.51 ± 1.66	111.15, 117.87
Max Long-Axis Rotation (°)	fore	0°	30.79 ± 1.86	27.03, 34.55	40.77 ± 1.84	22.40, 29.87	26.14 ± 1.84	37.03, 44.50	37.16 ± 1.84	33.43, 40.89
	fore	45°	25.53 ± 1.86	21.77, 29.29	37.14 ± 1.84	21.69, 29.16	25.42 ± 1.84	33.40, 40.87	33.93 ± 1.84	30.20, 37.66
	fore	80°	19.13 ± 1.86	15.37, 22.89	35.07 ± 1.88	8.36, 15.83	12.10 ± 1.84	31.26, 38.87	26.44 ± 1.84	22.71, 30.17
	fore	90°	15.18 ± 1.86	11.42, 18.94	29.06 ± 1.99	10.08, 30.28	20.18 ± 4.98	25.02, 33.10	19.00 ± 1.84	15.27, 22.73
	hind	0°	79.68 ± 1.86	75.92, 83.44	70.00 ± 1.84	70.25, 77.72	73.98 ± 1.84	66.27, 73.74	76.35 ± 1.84	72.63, 80.08
	hind	45°	82.85 ± 1.86	79.09, 86.61	67.92 ± 1.84	75.30, 82.77	79.04 ± 1.84	64.18, 71.65	83.45 ± 1.84	79.72, 87.18
	hind	80°	84.46 ± 1.86	80.70, 88.22	71.47 ± 1.88	73.89, 81.36	77.63 ± 1.84	67.66, 75.27	84.79 ± 1.84	81.06, 88.52
	hind	90°	82.54 ± 1.86	78.78, 86.30	70.86 ± 1.99	66.05, 86.25	76.15 ± 4.98	66.82, 74.90	82.96 ± 1.84	79.23, 86.69
Min Long-Axis Rotation (°)	fore	0°	-81.55 ± 1.76	-85.10, -78.00	-77.35 ± 1.74	-76.09, -69.03	-72.56 ± 1.74	-80.88, -73.81	-74.88 ± 1.74	-78.41, -71.35
	fore	45°	-80.08 ± 1.76	-83.63, -76.53	-76.91 ± 1.74	-76.51, -69.44	-72.97 ± 1.74	-80.44, -73.37	-77.16 ± 1.74	-80.69, -73.63
	fore	80°	-80.16 ± 1.76	-83.71, -76.61	-76.15 ± 1.79	-77.32, -70.26	-73.79 ± 1.74	-79.78, -72.52	-81.32 ± 1.74	-84.85, -77.79
	fore	90°	-79.68 ± 1.76	-83.23, -76.13	-75.33 ± 1.95	-83.57, -60.81	-72.19 ± 5.61	-79.29, -71.38	-79.81 ± 1.74	-83.34, -76.28
	hind	0°	5.85 ± 1.76	2.30, 9.40	-9.14 ± 1.74	-7.58, -0.51	-4.05 ± 1.74	-12.67, -5.60	-13.91 ± 1.74	-17.44, -10.38
	hind	45°	8.43 ± 1.76	4.88, 11.98	-12.08 ± 1.74	-0.11, 6.96	3.42 ± 1.74	-15.61, -8.54	-1.73 ± 1.74	-5.26, 1.80
	hind	80°	8.14 ± 1.76	4.59, 11.69	-9.67 ± 1.79	0.31, 7.38	3.84 ± 1.74	-13.30, -6.03	-13.07 ± 1.74	-16.60, -9.54
	hind	90°	11.79 ± 1.76	8.24, 15.34	-5.85 ± 1.95	-9.93, 12.83	1.45 ± 5.61	-9.80, -1.89	-11.07 ± 1.74	-14.60, -7.53
Long-Axis Rotation Excursion (°)	fore	0°	112.36 ± 2.40	107.50, 117.22	118.10 ± 2.38	93.88, 103.54	98.71 ± 2.38	113.27, 122.93	112.04 ± 2.38	107.21, 116.86
	fore	45°	105.64 ± 2.40	100.78, 110.49	114.03 ± 2.38	93.58, 103.24	98.41 ± 2.38	109.20, 118.87	111.08 ± 2.38	106.26, 115.91
	fore	80°	99.32 ± 2.40	94.46, 104.18	111.20 ± 2.44	81.07, 90.73	85.90 ± 2.38	106.25, 116.15	107.75 ± 2.38	102.92, 112.58
	fore	90°	94.88 ± 2.40	90.02, 99.74	104.37 ± 2.64	77.50, 107.23	92.37 ± 7.33	99.01, 109.72	98.81 ± 2.38	93.98, 103.63
	hind	0°	73.86 ± 2.40	69.00, 78.71	79.13 ± 2.38	73.21, 82.87	78.04 ± 2.38	74.30, 83.96	90.26 ± 2.38	85.43, 95.08
	hind	45°	74.45 ± 2.40	69.59, 79.30	79.99 ± 2.38	70.80, 80.46	75.63 ± 2.38	75.15, 84.82	85.18 ± 2.38	80.35, 90.00

	hind	80°	76.35 ± 2.40	71.49, 81.20	81.11 ± 2.44	68.97, 78.63	73.80 ± 2.38	76.16, 86.06	97.86 ± 2.38	93.03, 102.68
	hind	90°	70.78 ± 2.40	65.92, 75.63	76.68 ± 2.64	59.84, 89.57	74.70 ± 7.33	71.33, 82.03	94.02 ± 2.38	89.20, 98.85
Max Elbow/Knee (°)	fore	0°	140.65 ± 1.19	138.26, 143.05	138.83 ± 1.17	123.31, 128.07	125.69 ± 1.17	136.45, 141.21	136.60 ± 1.17	134.22, 138.98
	fore	45°	139.23 ± 1.19	136.84, 141.63	137.74 ± 1.17	129.19, 133.95	131.57 ± 1.17	135.36, 140.12	138.16 ± 1.17	135.79, 140.54
	fore	80°	140.32 ± 1.19	137.92, 142.72	140.56 ± 1.20	127.50, 132.27	129.88 ± 1.17	138.13, 143.00	137.69 ± 1.17	135.31, 140.07
	fore	90°	141.71 ± 1.19	139.32, 144.11	143.18 ± 1.28	122.36, 135.92	129.14 ± 3.34	140.58, 145.78	136.12 ± 1.17	133.74, 138.50
	hind	0°	149.57 ± 1.19	147.18, 151.97	156.46 ± 1.17	146.66, 151.42	149.04 ± 1.17	154.07, 158.84	151.59 ± 1.17	149.21, 153.97
	hind	45°	150.01 ± 1.19	147.61, 152.40	156.44 ± 1.17	148.31, 153.08	150.69 ± 1.17	154.06, 158.82	152.22 ± 1.17	149.84, 154.60
	hind	80°	153.62 ± 1.19	151.23, 156.02	159.93 ± 1.20	147.65, 152.41	150.03 ± 1.17	157.50, 162.37	151.69 ± 1.17	149.31, 154.07
	hind	90°	155.97 ± 1.19	153.58, 158.37	159.46 ± 1.28	148.33, 161.89	155.11 ± 3.34	156.86, 162.06	151.08 ± 1.17	148.70, 153.46
Min Elbow/Knee (°)	fore	0°	81.39 ± 2.07	77.21, 85.58	75.37 ± 2.05	59.08, 67.39	63.24 ± 2.05	71.22, 79.53	66.68 ± 2.05	62.53, 70.84
	fore	45°	82.79 ± 2.07	78.60, 86.98	76.64 ± 2.05	65.71, 74.03	69.87 ± 2.05	72.48, 80.80	72.63 ± 2.05	68.48, 76.78
	fore	80°	76.40 ± 2.07	72.22, 80.59	71.18 ± 2.09	60.61, 68.93	64.77 ± 2.05	66.95, 75.42	62.24 ± 2.05	58.09, 66.39
	fore	90°	75.94 ± 2.07	71.75, 80.12	73.24 ± 2.22	64.56, 86.92	75.74 ± 5.51	68.75, 77.74	63.65 ± 2.05	59.49, 67.80
	hind	0°	76.41 ± 2.07	72.22, 80.60	82.85 ± 2.05	74.37, 82.69	78.53 ± 2.05	78.69, 87.01	68.95 ± 2.05	64.79, 73.10
	hind	45°	72.91 ± 2.07	68.72, 77.10	73.28 ± 2.05	74.35, 82.67	78.51 ± 2.05	69.12, 77.44	67.85 ± 2.05	63.70, 72.01
	hind	80°	65.55 ± 2.07	61.36, 69.74	65.91 ± 2.09	66.73, 75.04	70.88 ± 2.05	61.68, 70.15	60.77 ± 2.05	56.62, 64.92
	hind	90°	73.15 ± 2.07	68.96, 77.34	72.06 ± 2.22	64.24, 86.60	75.42 ± 5.51	67.57, 76.55	66.39 ± 2.05	62.23, 70.54
Elbow/Knee Excursion (°)	fore	0°	59.27 ± 2.08	55.06, 63.48	63.45 ± 2.06	58.27, 66.64	62.46 ± 2.06	59.27, 67.64	69.92 ± 2.06	65.74, 74.10
	fore	45°	56.46 ± 2.08	52.24, 60.67	61.09 ± 2.06	57.52, 65.89	61.71 ± 2.06	56.91, 65.28	65.53 ± 2.06	61.35, 69.71
	fore	80°	63.93 ± 2.08	59.71, 68.14	69.37 ± 2.11	60.93, 69.30	65.12 ± 2.06	65.09, 73.64	75.45 ± 2.06	71.27, 79.63
	fore	90°	65.79 ± 2.08	61.58, 70.00	69.92 ± 2.25	41.62, 65.28	53.45 ± 5.83	65.36, 74.48	72.47 ± 2.06	68.29, 76.65
	hind	0°	73.17 ± 2.08	68.96, 77.38	73.60 ± 2.06	66.33, 74.70	70.51 ± 2.06	69.42, 77.79	82.64 ± 2.06	78.46, 86.82
	hind	45°	77.11 ± 2.08	72.90, 81.32	83.15 ± 2.06	68.00, 76.37	72.19 ± 2.06	78.97, 87.34	84.36 ± 2.06	80.18, 88.54
	hind	80°	88.08 ± 2.08	83.87, 92.29	94.01 ± 2.11	74.97, 83.34	79.15 ± 2.06	89.73, 98.28	90.92 ± 2.06	86.74, 95.10
	hind	90°	82.83 ± 2.08	78.62, 87.05	87.38 ± 2.25	67.91, 91.57	79.74 ± 5.83	82.82, 91.94	84.69 ± 2.06	80.51, 88.87
Max Wrist/ Ankle (°)	fore	0°	175.02 ± 0.81	173.37, 176.66	172.52 ± 0.81	169.52, 172.79	171.16 ± 0.81	170.88, 174.16	173.80 ± 0.81	172.17, 175.44
	fore	45°	172.40 ± 0.81	170.75, 174.04	171.43 ± 0.81	170.31, 173.58	171.95 ± 0.81	169.79, 173.07	174.77 ± 0.81	173.13, 176.40
	fore	80°	169.87 ± 0.81	168.22, 171.51	171.71 ± 0.83	169.36, 172.63	171.00 ± 0.81	170.03, 173.39	172.19 ± 0.81	170.55, 173.83
	fore	90°	168.39 ± 0.81	166.74, 170.03	171.36 ± 0.89	168.04, 178.10	173.07 ± 2.48	169.54, 173.17	172.12 ± 0.81	170.48, 173.75
	hind	0°	167.26 ± 0.81	165.62, 168.91	173.06 ± 0.81	159.66, 162.93	161.29 ± 0.81	171.42, 174.70	173.28 ± 0.81	171.64, 174.91
	hind	45°	166.39 ± 0.81	164.74, 168.04	170.11 ± 0.81	164.36, 167.63	165.99 ± 0.81	168.48, 171.75	172.82 ± 0.81	171.18, 174.45
	hind	80°	162.38 ± 0.81	160.73, 164.03	169.43 ± 0.83	158.91, 162.19	160.55 ± 0.81	167.75, 171.11	167.81 ± 0.81	166.18, 169.45
	hind	90°	162.65 ± 0.81	161.00, 164.29	166.35 ± 0.89	155.54, 165.60	160.57 ± 2.48	164.53, 168.16	166.36 ± 0.81	164.73, 168.00
	fore	0°	87.80 ± 2.03	83.68, 91.91	84.54 ± 2.02	93.62, 101.81	97.71 ± 2.02	80.44, 88.63	90.47 ± 2.02	86.37, 94.56

Journal of Experimental Biology • Supplementary information

Min Wrist/Ankle (°)	fore	45°	85.70 ± 2.03	81.59, 89.82	79.40 ± 2.02	91.18, 99.37	95.28 ± 2.02	75.30, 83.49	95.50 ± 2.02	91.41, 99.59
	fore	80°	86.53 ± 2.03	82.42, 90.64	84.05 ± 2.08	91.76, 99.95	95.86 ± 2.02	79.84, 88.27	90.13 ± 2.02	86.04, 94.22
	fore	90°	91.43 ± 2.03	87.31, 95.54	85.97 ± 2.27	84.61, 111.43	98.02 ± 6.61	81.38, 90.57	94.71 ± 2.02	90.62, 98.80
	hind	0°	67.89 ± 2.03	63.77, 72.00	71.76 ± 2.02	73.09, 81.28	77.19 ± 2.02	67.67, 75.86	77.65 ± 2.02	73.56, 81.74
	hind	45°	66.30 ± 2.03	62.19, 70.41	67.60 ± 2.02	65.40, 73.59	69.49 ± 2.02	63.50, 71.69	77.14 ± 2.02	73.05, 81.23
	hind	80°	64.40 ± 2.03	60.29, 68.52	71.51 ± 2.08	68.60, 76.79	72.70 ± 2.02	67.30, 75.72	70.27 ± 2.02	66.18, 74.36
	hind	90°	67.54 ± 2.03	63.42, 71.65	73.82 ± 2.27	57.36, 84.18	70.77 ± 6.61	69.22, 78.42	71.64 ± 2.02	67.55, 75.73
Wrist/Ankle Excursion (°)	fore	0°	87.22 ± 2.20	82.77, 91.67	87.98 ± 2.19	69.01, 77.88	73.44 ± 2.19	83.55, 92.41	83.34 ± 2.18	78.91, 87.76
	fore	45°	86.70 ± 2.20	82.24, 91.15	92.03 ± 2.19	72.24, 81.10	76.67 ± 2.19	87.60, 96.46	79.27 ± 2.18	74.84, 83.69
	fore	80°	83.34 ± 2.20	78.88, 87.79	87.68 ± 2.24	70.71, 79.57	75.14 ± 2.19	83.13, 92.23	82.06 ± 2.18	77.63, 86.49
	fore	90°	76.96 ± 2.20	72.51, 81.41	85.40 ± 2.44	61.01, 89.10	75.06 ± 6.93	80.46, 90.34	77.41 ± 2.18	72.98, 81.84
	hind	0°	99.38 ± 2.20	94.93, 103.83	101.30 ± 2.19	79.68, 88.54	84.11 ± 2.19	96.86, 105.73	95.63 ± 2.18	91.20, 100.05
	hind	45°	100.09 ± 2.20	95.64, 104.55	102.51 ± 2.19	92.07, 100.93	96.50 ± 2.19	98.08, 106.95	95.68 ± 2.18	91.25, 100.11
	hind	80°	97.98 ± 2.20	93.52, 102.43	97.94 ± 2.24	83.42, 92.28	87.85 ± 2.19	93.39, 102.49	97.55 ± 2.18	93.12, 101.97
	hind	90°	95.11 ± 2.20	90.66, 99.57	92.55 ± 2.44	75.76, 103.85	89.81 ± 6.93	87.60, 97.49	94.72 ± 2.18	90.29, 99.15
Max Contralateral Girdle Rotation (°)	fore	0°	17.59 ± 0.73	16.12, 19.07	19.78 ± 0.72	11.54, 14.48	13.01 ± 0.72	18.31, 21.25	19.32 ± 0.72	17.85, 20.79
	fore	45°	19.71 ± 0.73	18.24, 21.19	24.53 ± 0.72	12.39, 15.33	13.86 ± 0.72	23.06, 26.00	21.71 ± 0.72	20.24, 23.18
	fore	80°	16.01 ± 0.73	14.53, 17.48	19.36 ± 0.75	10.73, 13.67	12.20 ± 0.72	17.84, 20.88	13.23 ± 0.72	11.76, 14.70
	fore	90°	16.37 ± 0.73	14.90, 17.85	16.87 ± 0.82	8.89, 19.11	14.00 ± 2.52	15.20, 18.54	12.52 ± 0.72	11.06, 13.99
	hind	0°	20.06 ± 0.73	18.59, 21.54	18.62 ± 0.72	7.73, 10.67	9.20 ± 0.72	17.15, 20.09	14.45 ± 0.72	12.99, 15.92
	hind	45°	22.19 ± 0.73	20.72, 23.67	18.23 ± 0.72	8.83, 11.77	10.30 ± 0.72	16.76, 19.70	16.21 ± 0.72	14.74, 17.68
	hind	80°	19.57 ± 0.73	18.10, 21.05	18.55 ± 0.75	9.27, 12.21	10.74 ± 0.72	17.03, 20.06	16.08 ± 0.72	14.61, 17.55
	hind	90°	20.50 ± 0.73	19.02, 21.97	16.97 ± 0.82	2.64, 12.86	7.75 ± 2.52	15.30, 18.65	14.54 ± 0.72	13.07, 16.01
Max Ipsilateral Girdle Rotation (°)	fore	0°	-19.41 ± 0.91	-21.26, -17.57	-21.51 ± 0.90	-15.20, -11.53	-13.37 ± 0.90	-23.34, -19.67	-21.72 ± 0.90	-23.55, -19.89
	fore	45°	-20.37 ± 0.91	-22.21, -18.52	-23.24 ± 0.90	-16.05, -12.38	-14.21 ± 0.90	-25.07, -21.40	-23.79 ± 0.90	-25.63, -21.96
	fore	80°	-18.31 ± 0.91	-20.15, -16.46	-21.34 ± 0.93	-15.84, -12.17	-14.01 ± 0.90	-23.22, -19.46	-19.34 ± 0.90	-21.18, -17.51
	fore	90°	-18.10 ± 0.91	-19.94, -16.25	-19.92 ± 1.00	-12.34, -1.33	-6.83 ± 2.71	-21.94, -17.90	-16.60 ± 0.90	-18.44, -14.77
	hind	0°	-17.13 ± 0.91	-18.98, -15.29	-21.05 ± 0.90	-12.19, -8.52	-10.35 ± 0.90	-22.88, -19.21	-15.12 ± 0.90	-16.96, -13.29
	hind	45°	-19.63 ± 0.91	-21.48, -17.79	-21.93 ± 0.90	-15.32, -11.65	-13.48 ± 0.90	-23.77, -20.10	-18.60 ± 0.90	-20.44, -16.77
	hind	80°	-19.20 ± 0.91	-21.04, -17.35	-24.00 ± 0.93	-14.83, -11.16	-13.00 ± 0.90	-25.88, -22.12	-18.19 ± 0.90	-20.03, -16.36
	hind	90°	-19.36 ± 0.91	-21.20, -17.51	-21.00 ± 1.00	-21.32, -10.31	-15.81 ± 2.71	-23.03, -18.98	-15.96 ± 0.90	-17.80, -14.13
Girdle Excursion (°)	fore	0°	36.99 ± 1.26	34.43, 39.55	41.29 ± 1.25	23.83, 28.91	26.37 ± 1.25	38.75, 43.83	41.05 ± 1.25	38.50, 43.59
	fore	45°	40.07 ± 1.26	37.51, 42.62	47.78 ± 1.25	25.53, 30.61	28.07 ± 1.25	45.23, 50.32	45.50 ± 1.25	42.96, 48.04
	fore	80°	34.30 ± 1.26	31.74, 36.86	40.64 ± 1.28	23.66, 28.75	26.21 ± 1.25	38.04, 43.25	32.57 ± 1.25	30.03, 35.12
	fore	90°	34.46 ± 1.26	31.90, 37.02	36.70 ± 1.38	13.15, 28.43	20.79 ± 3.77	33.90, 39.51	29.13 ± 1.25	26.59, 31.67

Journal of Experimental Biology • Supplementary information

	hind	0°	37.19 ± 1.26	34.63, 39.75	39.67 ± 1.25	17.00, 22.09	19.55 ± 1.25	37.13, 42.22	29.58 ± 1.25	27.04, 32.12
	hind	45°	41.81 ± 1.26	39.26, 44.37	40.17 ± 1.25	21.23, 26.32	23.77 ± 1.25	37.63, 42.71	34.82 ± 1.25	32.28, 37.36
	hind	80°	38.76 ± 1.26	36.20, 41.32	42.49 ± 1.28	21.19, 26.27	23.73 ± 1.25	39.89, 45.10	34.28 ± 1.25	31.73, 36.82
	hind	90°	39.85 ± 1.26	37.29, 42.40	37.88 ± 1.38	15.88, 31.16	23.52 ± 3.77	35.08, 40.69	30.50 ± 1.25	27.96, 33.05
Foot Angle (°)	fore	0°	14.62 ± 2.12	10.34, 18.89	7.28 ± 2.10	-20.02, -11.52	-15.77 ± 2.10	3.03, 11.54	-0.74 ± 2.09	-4.99, 3.51
	fore	45°	15.19 ± 2.12	10.91, 19.47	11.92 ± 2.10	-9.12, -0.62	-4.87 ± 2.10	7.67, 16.17	8.83 ± 2.09	4.58, 13.08
	fore	80°	18.85 ± 2.12	14.57, 23.13	14.06 ± 2.14	-3.81, 4.70	0.45 ± 2.10	9.71, 18.41	11.31 ± 2.09	7.07, 15.56
	fore	90°	23.95 ± 2.12	19.67, 28.22	18.28 ± 2.30	-6.87, 17.87	5.50 ± 6.10	13.62, 22.94	11.29 ± 2.09	7.04, 15.53
	hind	0°	-4.32 ± 2.12	-8.60, -0.04	5.70 ± 2.10	-8.41, 0.09	-4.16 ± 2.10	1.45, 9.95	-11.11 ± 2.09	-15.36, -6.86
	hind	45°	-6.68 ± 2.12	-10.96, -2.40	3.53 ± 2.10	-13.83, -5.32	-9.58 ± 2.10	-0.73, 7.78	-6.38 ± 2.09	-10.63, -2.14
	hind	80°	9.15 ± 2.12	4.87, 13.43	11.13 ± 2.14	3.81, 12.31	8.06 ± 2.10	6.79, 15.48	4.42 ± 2.09	0.18, 8.67
	hind	90°	26.96 ± 2.12	22.68, 31.24	18.74 ± 2.30	-11.72, 13.02	0.65 ± 6.10	14.08, 23.40	5.95 ± 2.09	1.70, 10.20
Digital Spread (°)	Fore	0°	105.83 ± 1.58	102.64, 109.02	107.06 ± 1.56	100.65, 106.97	103.81 ± 1.56	103.90, 110.22	109.24 ± 1.55	106.09, 112.40
	Fore	45°	102.16 ± 1.58	98.97, 105.34	104.64 ± 1.56	97.09, 103.41	100.25 ± 1.56	101.48, 107.80	106.96 ± 1.55	103.81, 110.11
	Fore	80°	102.52 ± 1.58	99.33, 105.70	100.12 ± 1.58	91.28, 97.60	94.44 ± 1.56	96.92, 103.31	101.18 ± 1.56	98.03, 104.34
	Fore	90°	99.86 ± 1.58	96.67, 103.04	98.46 ± 1.64	89.03, 103.20	96.12 ± 3.49	95.13, 101.79	101.09 ± 1.56	97.93, 104.24
	Hind	0°	117.02 ± 1.58	113.83, 120.21	120.01 ± 1.56	110.38, 116.69	113.53 ± 1.56	116.85, 123.17	114.92 ± 1.55	111.76, 118.07
	Hind	45°	116.61 ± 1.58	113.42, 119.80	117.20 ± 1.56	105.42, 111.74	108.58 ± 1.56	114.04, 120.36	111.14 ± 1.55	107.99, 114.29
	Hind	80°	118.14 ± 1.58	114.95, 121.32	117.46 ± 1.58	96.82, 103.14	99.98 ± 1.56	114.27, 120.66	105.53 ± 1.56	102.37, 108.68
	Hind	90°	114.79 ± 1.58	111.61, 117.98	114.40 ± 1.64	89.15, 103.33	96.24 ± 3.49	111.08, 117.73	106.38 ± 1.56	103.23, 109.54
Limb Spread (SVL)	fore	0°	0.093 ± 0.003	0.087, 0.099	0.066 ± 0.003	0.062, 0.074	0.068 ± 0.003	0.060, 0.072	0.087 ± 0.003	0.081, 0.093
	fore	45°	0.095 ± 0.003	0.089, 0.101	0.074 ± 0.003	0.064, 0.076	0.070 ± 0.003	0.068, 0.080	0.088 ± 0.003	0.082, 0.094
	fore	80°	0.088 ± 0.003	0.083, 0.094	0.075 ± 0.003	0.060, 0.071	0.066 ± 0.003	0.069, 0.081	0.089 ± 0.003	0.084, 0.095
	fore	90°	0.098 ± 0.003	0.092, 0.104	0.084 ± 0.003	0.061, 0.095	0.078 ± 0.008	0.078, 0.091	0.092 ± 0.003	0.086, 0.098
	hind	0°	0.124 ± 0.003	0.119, 0.130	0.108 ± 0.003	0.089, 0.101	0.095 ± 0.003	0.102, 0.114	0.105 ± 0.003	0.099, 0.111
	hind	45°	0.125 ± 0.003	0.119, 0.131	0.109 ± 0.003	0.083, 0.095	0.089 ± 0.003	0.103, 0.115	0.099 ± 0.003	0.093, 0.105
	hind	80°	0.122 ± 0.003	0.116, 0.128	0.103 ± 0.003	0.088, 0.100	0.094 ± 0.003	0.097, 0.109	0.090 ± 0.003	0.084, 0.096
	hind	90°	0.135 ± 0.003	0.129, 0.140	0.109 ± 0.003	0.061, 0.095	0.078 ± 0.008	0.103, 0.116	0.096 ± 0.003	0.091, 0.102
Max Hip Height (SVL)	fore	0°	0.112 ± 0.005	0.103, 0.121	0.131 ± 0.005	0.075, 0.093	0.084 ± 0.005	0.122, 0.140	0.107 ± 0.005	0.098, 0.116
	fore	45°	0.088 ± 0.005	0.078, 0.097	0.104 ± 0.005	0.062, 0.080	0.071 ± 0.005	0.094, 0.113	0.095 ± 0.005	0.086, 0.104
	fore	80°	0.074 ± 0.005	0.065, 0.084	0.098 ± 0.005	0.060, 0.078	0.069 ± 0.005	0.088, 0.107	0.092 ± 0.005	0.083, 0.101
	fore	90°	0.067 ± 0.005	0.058, 0.076	0.082 ± 0.005	0.051, 0.092	0.072 ± 0.010	0.072, 0.092	0.077 ± 0.005	0.067, 0.086
	hind	0°	0.094 ± 0.005	0.085, 0.104	0.113 ± 0.005	0.058, 0.076	0.067 ± 0.005	0.104, 0.122	0.105 ± 0.005	0.096, 0.114
	hind	45°	0.081 ± 0.005	0.072, 0.091	0.088 ± 0.005	0.048, 0.067	0.057 ± 0.005	0.079, 0.097	0.080 ± 0.005	0.071, 0.089
	hind	80°	0.069 ± 0.005	0.060, 0.078	0.090 ± 0.005	0.054, 0.072	0.063 ± 0.005	0.081, 0.099	0.073 ± 0.005	0.064, 0.083

Journal of Experimental Biology • Supplementary information

	hind	90°	0.061 ± 0.005	0.052, 0.070	0.084 ± 0.005	0.052, 0.093	0.073 ± 0.010	0.075, 0.094	0.060 ± 0.005	0.051, 0.069
Min Hip Height (SVL)	fore	0°	0.063 ± 0.004	0.055, 0.071	0.063 ± 0.004	0.037, 0.053	0.045 ± 0.004	0.055, 0.071	0.064 ± 0.004	0.056, 0.072
	fore	45°	0.050 ± 0.004	0.042, 0.058	0.055 ± 0.004	0.030, 0.046	0.038 ± 0.004	0.047, 0.064	0.051 ± 0.004	0.043, 0.060
	fore	80°	0.041 ± 0.004	0.033, 0.049	0.054 ± 0.004	0.031, 0.047	0.039 ± 0.004	0.046, 0.062	0.048 ± 0.004	0.040, 0.056
	fore	90°	0.034 ± 0.004	0.026, 0.042	0.044 ± 0.004	0.028, 0.066	0.047 ± 0.009	0.036, 0.053	0.034 ± 0.004	0.026, 0.042
	hind	0°	0.063 ± 0.004	0.055, 0.071	0.080 ± 0.004	0.036, 0.052	0.044 ± 0.004	0.072, 0.088	0.073 ± 0.004	0.064, 0.081
	hind	45°	0.056 ± 0.004	0.047, 0.064	0.059 ± 0.004	0.031, 0.047	0.039 ± 0.004	0.051, 0.067	0.056 ± 0.004	0.047, 0.064
	hind	80°	0.047 ± 0.004	0.039, 0.055	0.061 ± 0.004	0.038, 0.054	0.046 ± 0.004	0.053, 0.069	0.052 ± 0.004	0.044, 0.060
	hind	90°	0.038 ± 0.004	0.030, 0.046	0.058 ± 0.004	0.041, 0.079	0.060 ± 0.009	0.050, 0.067	0.040 ± 0.004	0.032, 0.048
Hip Excursion (SVL)	fore	0°	0.049 ± 0.002	0.045, 0.053	0.068 ± 0.002	0.035, 0.043	0.039 ± 0.002	0.064, 0.072	0.043 ± 0.002	0.040, 0.047
	fore	45°	0.038 ± 0.002	0.034, 0.041	0.048 ± 0.002	0.029, 0.037	0.033 ± 0.002	0.044, 0.052	0.044 ± 0.002	0.040, 0.048
	fore	80°	0.033 ± 0.002	0.029, 0.037	0.043 ± 0.002	0.026, 0.034	0.030 ± 0.002	0.039, 0.047	0.044 ± 0.002	0.040, 0.048
	fore	90°	0.033 ± 0.002	0.029, 0.037	0.038 ± 0.002	0.014, 0.036	0.025 ± 0.006	0.033, 0.042	0.043 ± 0.002	0.039, 0.047
	hind	0°	0.032 ± 0.002	0.028, 0.035	0.033 ± 0.002	0.019, 0.026	0.023 ± 0.002	0.029, 0.037	0.033 ± 0.002	0.029, 0.037
	hind	45°	0.026 ± 0.002	0.022, 0.030	0.029 ± 0.002	0.015, 0.022	0.019 ± 0.002	0.025, 0.033	0.025 ± 0.002	0.021, 0.029
	hind	80°	0.022 ± 0.002	0.018, 0.026	0.029 ± 0.002	0.013, 0.021	0.017 ± 0.002	0.025, 0.033	0.021 ± 0.002	0.017, 0.025
	hind	90°	0.023 ± 0.002	0.019, 0.027	0.026 ± 0.002	0.002, 0.024	0.013 ± 0.006	0.022, 0.030	0.020 ± 0.002	0.016, 0.024

Journal of Experimental Biology • Supplementary information

Table S3. Coefficients of linear discriminant (LD) axes from the discriminant function analysis performed on limb kinematics. The first 14 out of 23 total linear discriminant axes are shown, representing 99% of the total variance.

**Variable**	**LD1**	**LD2**	**LD3**	**LD4**	**LD5**	**LD6**	**LD7**	**LD8**	**LD9**	**LD10**	**LD11**	**LD12**	**LD13**	**LD14**
Max Abduction	0.24	0.54	-0.61	0.12	-0.03	-0.70	0.45	0.13	1.09	-0.85	-0.14	0.80	-0.06	-0.75
Max Adduction	-0.03	0.13	-0.02	0.21	0.10	0.02	-0.24	0.02	-0.13	-0.10	-0.05	0.03	0.43	0.34
Abduction/Adduction Excursion	-0.27	-0.03	0.55	-0.08	-0.08	0.43	0.16	0.59	-0.86	1.04	0.76	-0.41	0.04	0.85
Max Protraction	0.34	-0.21	0.27	0.02	-0.19	1.13	3.21	-2.45	-1.08	-1.47	-0.88	-0.10	0.63	0.16
Max Retraction	-0.04	-0.01	0.51	-0.59	-0.02	0.82	1.64	-1.23	-0.25	-1.46	-1.84	-0.06	0.72	0.00
Protraction/Retraction Excursion	0.33	0.50	-0.95	-0.63	0.44	-0.84	-3.37	1.76	0.62	1.96	1.96	-0.16	-0.74	-0.30
Max Anterior Long-Axis Rotation	0.77	0.14	-0.23	0.57	0.64	0.77	0.22	0.83	0.36	-0.33	-0.03	0.72	-0.43	-0.24
Max Posterior Long-Axis Rotation	0.03	-0.09	-0.07	-0.05	-0.06	0.01	0.13	0.07	-0.15	0.05	-0.32	0.37	-0.44	0.02
Long-Axis Rotation Excursion	0.00	-0.20	-0.06	-0.55	-0.15	-1.00	0.06	-0.04	-0.57	0.04	-0.02	-0.28	0.21	0.46
Max Elbow/Knee Extension	-0.36	0.33	0.57	-0.46	0.27	-0.15	0.09	0.02	0.03	0.23	0.45	0.12	0.53	0.04
Max Elbow/Knee Flexion	0.18	0.09	0.31	-0.10	-0.08	-0.54	-0.10	0.16	0.24	0.23	0.10	-0.63	0.21	-0.13
Elbow/Knee Excursion	0.30	-0.23	-0.41	0.45	-0.08	0.76	0.41	-0.09	0.16	0.07	-0.18	0.18	-0.09	0.19
Max Wrist/Ankle Extension	0.01	0.16	0.31	0.23	-0.31	0.25	-0.40	-0.11	0.41	-1.59	0.76	0.39	-0.48	1.47
Max Wrist/Ankle Flexion	-0.12	0.31	-0.20	0.29	-0.13	0.00	-0.32	0.14	0.13	-1.65	1.27	0.33	-0.58	1.81
Wrist/Ankle Excursion	0.30	-1.46	-0.37	-0.89	0.20	-0.20	0.80	-0.36	0.34	2.88	-1.19	-0.55	0.61	-2.60
Max Contralateral Yaw	1.09	-0.04	0.92	0.36	0.57	-1.16	-0.34	-0.35	-0.09	0.15	0.06	0.56	0.88	-0.01
Max Ipsilateral Yaw	-0.49	-0.25	0.03	0.12	-0.56	-0.41	0.05	-0.31	0.02	-0.23	0.07	0.71	0.67	0.52
Max Yaw Excursion	-1.05	-0.07	-0.18	-0.40	0.41	0.19	-0.70	-0.25	-0.20	-0.34	0.33	-0.28	-0.90	-0.35
Foot Angle	-0.41	-0.68	-1.09	-0.06	0.68	-0.52	0.02	0.07	-0.23	-0.20	-0.21	-0.29	0.36	-0.08
Digital Spread	0.30	-0.25	0.35	-0.12	-0.08	-0.21	0.19	0.20	-0.01	-0.84	0.12	-0.61	-0.16	0.07
Limb Spread	0.61	-0.18	0.18	-0.88	-1.17	0.37	-0.48	-0.14	-0.59	0.17	0.36	0.52	-0.09	-0.28
Max Shoulder/Hip Height	0.04	-0.04	0.12	0.07	0.19	-0.07	0.10	-0.01	0.03	0.06	-0.39	0.21	-0.21	0.01
Min Shoulder/Hip Height	0.04	-0.04	0.12	0.07	0.19	-0.07	0.10	-0.01	0.03	0.06	-0.39	0.21	-0.21	0.01
Shoulder/Hip Excursion	-0.44	-0.03	0.44	0.30	0.11	-0.20	0.37	-0.09	-0.60	-0.20	0.40	-0.18	0.31	-0.81
Proportion of Trace	0.647	0.096	0.074	0.043	0.038	0.023	0.020	0.015	0.012	0.008	0.006	0.004	0.003	0.003
Cumulative Proportion	0.647	0.743	0.817	0.860	0.898	0.921	0.941	0.956	0.968	0.975	0.981	0.985	0.989	0.991

Journal of Experimental Biology • Supplementary information
